# Prediction of mosquito species and population age structure using mid-infrared spectroscopy and supervised machine learning

**DOI:** 10.12688/wellcomeopenres.15201.3

**Published:** 2019-09-16

**Authors:** Mario González Jiménez, Simon A. Babayan, Pegah Khazaeli, Margaret Doyle, Finlay Walton, Elliott Reedy, Thomas Glew, Mafalda Viana, Lisa Ranford-Cartwright, Abdoulaye Niang, Doreen J. Siria, Fredros O. Okumu, Abdoulaye Diabaté, Heather M. Ferguson, Francesco Baldini, Klaas Wynne

**Affiliations:** 1 School of Chemistry, University of Glasgow, Glasgow, G12 8QQ, UK; 2 Institute of Biodiversity Animal Health and Comparative Medicine, University of Glasgow, Glasgow, G12 8QQ, UK; 3 Department of Medical Biology and Public Health, Institut de Recherche en Science de la Santé (IRSS), Bobo-Dioulasso, Burkina Faso; 4 Environmental Health & Ecological Sciences Department, Ifakara Health Institute, Off Mlabani Passage, PO Box 53, Ifakara, Tanzania

**Keywords:** Malaria, Anopheles gambiae, Anopheles arabiensis, Vector control, Machine learning, Mid-infrared spectroscopy

## Abstract

Despite the global efforts made in the fight against malaria, the disease is resurging. One of the main causes is the resistance that *Anopheles* mosquitoes, vectors of the disease, have developed to insecticides. *Anopheles* must survive for at least 10 days to possibly transmit malaria. Therefore, to evaluate and improve malaria vector control interventions, it is imperative to monitor and accurately estimate the age distribution of mosquito populations as well as their population sizes. Here, we demonstrate a machine-learning based approach that uses mid-infrared spectra of mosquitoes to characterise simultaneously both age and species identity of females of the African malaria vector species *Anopheles gambiae* and *An. arabiensis*, using laboratory colonies. Mid-infrared spectroscopy-based prediction of mosquito age structures was statistically indistinguishable from true modelled distributions. The accuracy of classifying mosquitoes by species was 82.6%. The method has a negligible cost per mosquito, does not require highly trained personnel, is rapid, and so can be easily applied in both laboratory and field settings. Our results indicate this method is a promising alternative to current mosquito species and age-grading approaches, with further improvements to accuracy and expansion for use with wild mosquito vectors possible through collection of larger mid-infrared spectroscopy data sets.

Between 2000 and 2015, insecticide-based control interventions targeting mosquito vectors averted an estimated 537 million malaria cases ^1^. Nevertheless, malaria still kills hundreds of thousands of people each year (445,000 in 2016), mainly in sub-Saharan Africa ^2^. Additionally, there is concern that progress may have stalled after more than a decade of success in global malaria control ^2^. Of major concern is the increase in insecticide resistance among mosquito populations throughout Africa ^3^, which is degrading the lethality and effectiveness of vector control tools, notably indoor residual spraying (IRS) and long-lasting insecticide treated nests (LLINs) which have been the cornerstones of malaria control in the past decades ^4^. Indeed, much of the effectiveness of LLINs and IRS comes from community-wide reductions in vector population size, not merely from preventing people from getting bitten ^5^.

Measurement of female mosquito vector survival is an important biological determinant of malaria transmission intensity ^6, 7^. This is because malaria parasites ( *Plasmodium* spp.) require more than 10 days of incubation inside female mosquito vectors (extrinsic incubation period, EIP) before they become infectious ^8– 11^. While there is uncertainty about mosquito survival in the field, crude estimates suggest the median lifespan of African malaria vectors is 7–10 days ^12^. Thus, only relatively old mosquitoes can transmit the parasite ^13^. As a result, even minor reductions in mosquito survival can have exponential impacts on pathogen transmission ^10, 14^. Consequently, accurate and high-resolution estimation of both mosquito abundance and longevity is essential for the assessment of the impact of various vector control measures.

Despite the crucial importance of mosquito demography to vector control, there are few reliable tools for rapid, high-throughput monitoring of mosquito survival in the wild. Conventionally, mosquito age has been approximated by classifying females (the only sex that transmits malaria) into groups based on their reproductive status as assessed through observation of their ovarian tracheoles ^15^. This widely-employed technique distinguishes females who have not yet laid eggs (nulliparous) from those that have laid at least one egg batch (parous), with the latter group assumed to be older than the former because the gonotrophic cycle between blood feeding and oviposition takes ~ 4 days. While useful for approximating general patterns of survival ^16^, this method is crude and cannot distinguish between females who have laid eggs only once or multiple times. Alternatively, more refined methods have been developed to estimate the number of gonotrophic cycles a female mosquito has gone through based on follicular relics or dilatations formed during each oviposition ^17^, although the conversion between gonotrophic cycles and actual age is imprecise (especially now that LLINs are limiting regular access to blood-meals) ^18^. While an improvement on the simple parity classification method, this approach is extremely technically demanding and time-consuming ^19^. Additionally, it is unsuitable for analysis of the large sample sizes necessary for estimating mosquito population structure ^20^.

Given these problems with ovary-based assessment ^21^, there has been significant investigation of alternative, molecular-based approaches to estimate mosquito age. These methods include: counting cuticle rings representing daily growth layers of the mosquito skeletal apodemes ^22^, chromatographic analysis of cuticular hydrocarbon chains ^23^, assessment of pteridines using fluorescence techniques ^24^, transcriptomic profiling ^25^, and mass spectrometric analysis of mosquito protein expression ^26^. However, thus far the level of accuracy, high cost, and/or need of highly trained users suggest that they might not be suitable for application in the field.

In addition to age, identification of mosquito species is crucial for estimation of malaria transmission dynamics. In Africa, the bulk of malaria transmission is carried out by members of the *Anopheles gambiae* sensu latu and *Anopheles funestus* sensu latu species compleses ^27^. The *An. gambiae* s.l. complex includes several morphological identical sibling species that can only be distinguished by molecular analysis ^28– 30^. Despite being morphologically identical, members of this group vary significantly in behaviour, transmission potential, and response to vector control measurements ^31^. For example, two major vectors in the *An. gambiae* s.l. group, *An. arabiensis* and *An. gambiae*, can differ in their propensity to enter and rest in houses, their host species choice, breeding conditions, resistance to insecticides, and tolerance to dry climates ^6, 32, 33^. Currently, *An. gambiae* s.l. species are best distinguished by polymerase chain reaction (PCR) methods ^30, 34– 36^, which are time-consuming and still relatively costly, and can thus only be carried out on a subsample of mosquitoes collected during typical entomological surveillance conducted by many agencies in Africa. Alternative techniques have been developed such as isoenzyme electrophoresis ^37^ or chromatography of cuticular components ^24^, but these are also very laborious and have weak discriminatory power ^38^.

Non-PCR-based methods often rely on structural and chemical differences in the cuticle to discriminate insects according to their species and other traits. In particular, near-infrared spectroscopy (NIRS) has been evaluated as a general strategy for examining insects since it does not require reagents and holds promise as a fast, practical, non-destructive, and cost-effective method for entomological surveillance. The results obtained to date have proved that the chemical composition of mosquitoes and other insects not only changes between species ^39– 41^, also across different age ^40, 42– 45^, according to resistance to insecticides ^46^ and in the presence of an infectious agent ^47, 48^. While promising, the NIRS typical approach has certain drawbacks. As it employs the most energetic portion of the infrared spectrum, the absorption bands are generated by two indirect processes: overtones (a vibration excited at a multiple of the fundamental frequency) and combinations (two or more fundamental vibrations excited simultaneously). Both processes are more incoherent and less frequent than the absorption of light by fundamental vibrations, so their absorption bands are wide and weak. As a result, NIR spectrum of a mosquito, formed by a combination of dozens of these bands, consists of a few features standing out against a background of continuous absorption ^49^. Also, most NIRS analyses use a dispersive method to collect the absorption spectra from insects, so the reflectivity of the sample is not controlled and the intensity of the bands of the spectrum depends on how the mosquito is placed in the spectrometer. In addition, the results are normally analysed using Partial Least Squares (PLS) regression, which is prone to over-fitting (i.e. the production of a model that corresponds too closely to a particular set of data and may therefore fail to predict future observations reliably) ^50^. This problem commonly arises when the number of samples is relatively small, and the number of variables is large.

Here we tested if these limitations can be overcome by shifting the measurement range (25,000–4,000 cm ^-1^) to the mid-infrared region (4,000–400 cm ^-1^), employing an attenuated total reflectance (ATR) device to assess the mosquitoes, and modelling the results with supervised machine learning. The mid-infrared absorption spectrum of a mosquito contains a set of discrete well-delineated bands that depend on the fundamental vibrations of the molecules present in the cuticle, providing a wealth of information not present in the near-infrared range, where it is not possible to capture the contributions of different biochemical components of the mosquito to the spectrum and their variations among mosquitoes with different attributes, as shown in *Aedes aegypti* and the diptera *Culicoides sonorensis* ^51, 52^. However, since the mid-infrared spectral bands are affected in non-trivial ways by the development of a mosquito and the changing composition of the cuticle, it is not possible to predict traits by simply monitoring changes in band intensities ^51^.

Here, we show that the use of supervised machine learning ^53^ allows the determination of the age and species of two major malaria vectors, *An. arabiensis* and *An. gambiae*, from the information contained in their mid-infrared spectra. This is possible because machine learning, unlike standard statistical approaches, can recognise the complex relationships in these traits (mosquito species and mosquito age) and disentangle them from other irrelevant variation ^54– 56^. Using this approach, we are able to reconstruct simulated age distributions of mosquito populations with unprecedented reliability. The technique we propose here is time efficient (an analysis takes less than one minute per mosquito), economical, and requires neither reagents nor highly trained operators. It also represents a novel approach to the analysis of insects using spectroscopic techniques, solving some previous drawbacks, and accelerating progress towards the establishment of infrared spectroscopy as a routine approach for mosquito surveillance and evaluation of interventions.

## Methods

### Mosquito rearing, blood feeding, and processing

*Anopheles gambiae* s.s (Kisumu strain) and *An. arabiensis* (Ifakara strain) mosquitoes were reared under standard insectary conditions of 27 ± 1°C, 70% humidity and a 12-hr light: 12-hr dark cycle at the University of Glasgow. *Anopheles gambiae* s.s (Kisumu strain) mosquitoes were provided by Hilary Ranson (Liverpool School of Tropical Medicine). The *An. arabiensis* (Ifakara strain) colony was initially established in 2008 at the Ifakara Health Institute with individuals from Sagamaganga village (Kilombero District, Morogoro Region, Tanzania) ^57^, and after a few generations reared at the University of Glasgow. Larvae were fed *ad libitum* on fish pellets (Tetra Pond Pellets, Tetra GmbH, Herrenteich 78, D49324). Pupae were collected from the larval trays and moved into a cage for emergence. Mosquitoes were considered to be in the age category of “Day 0” on their day of emergence from pupa to adult. Upon emergence, adults were fed *ad libitum* on a 5% glucose solution supplemented with 0.05% (w/v) 4-aminobenzoic acid (PABA).

In order to produce mosquitoes with the same age and different physiological conditions, cages with mosquitoes of the same age (where pupae were added on the same day) were blood fed human blood and membrane feeders at different days after emergence. An oviposition cup was then introduced 2 days after a blood meal to allow egg laying. Mosquitoes under three types of physiological conditions were collected, specifically: mosquitoes that had just received a blood meal (blood fed), mosquitoes that developed eggs as they received a blood meal two days before collection (gravid) and mosquitoes that laid eggs as they received a blood meal four days before collection and had the chance to lay eggs on an oviposition cup for two consecutive nights (sugar fed). Blood feeding was provided to each cage every 6 days. Thus, mosquitoes living 6 or more days after their first blood meal underwent multiple gonotrophic cycles.

Human blood was obtained from the Glasgow and West of Scotland Blood Transfusion Service. Ethical approval for the supply and use of human blood was obtained from Scottish National Blood Transfusion Service committee for governance of blood and tissue samples for non-therapeutic use, and Donor Research (submission Reference No 18~15). Whole blood from donors of any blood group was provided in Citrate-Phosphate-Dextrose-Adenine (CPD-A) anticoagulant/preservative. Fresh blood was obtained on a weekly basis.

Upon collection, mosquitoes were transferred into a cup and killed with a cotton soaked with chloroform placed on top of the cup for 30 minutes. Dead mosquitoes were then transferred into a tube over a layer of cotton and silica gel desiccant. The vial was then immediately stored at 4°C. Since it takes one day to dry in silica *Anopheles gambiae* mosquitoes and two days *An. arabiensis*, both species were stored prior to measurement for at least three days.

### Spectral data acquisition

**Figure 1.**
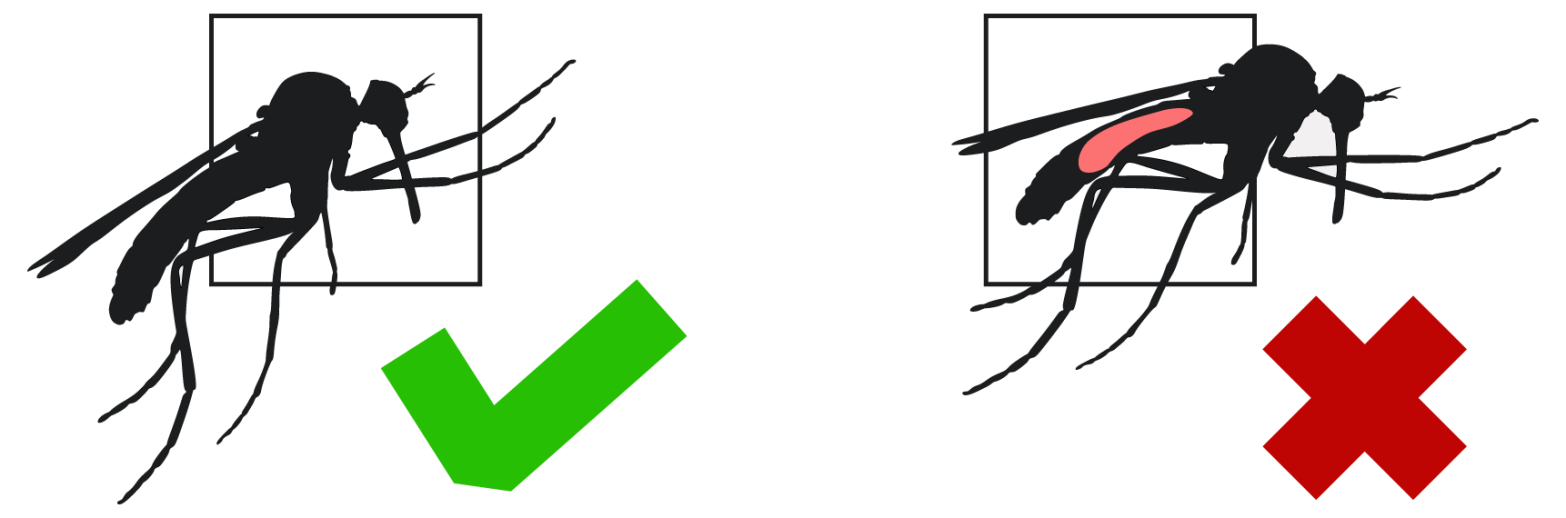
Best position of the mosquito on the ATR crystal. The correct way to place a mosquito on the ATR crystal (left) is to cover the surface with the head and chest. The wrong way (right) is by centring the abdomen on the crystal.

**Figure 2.**
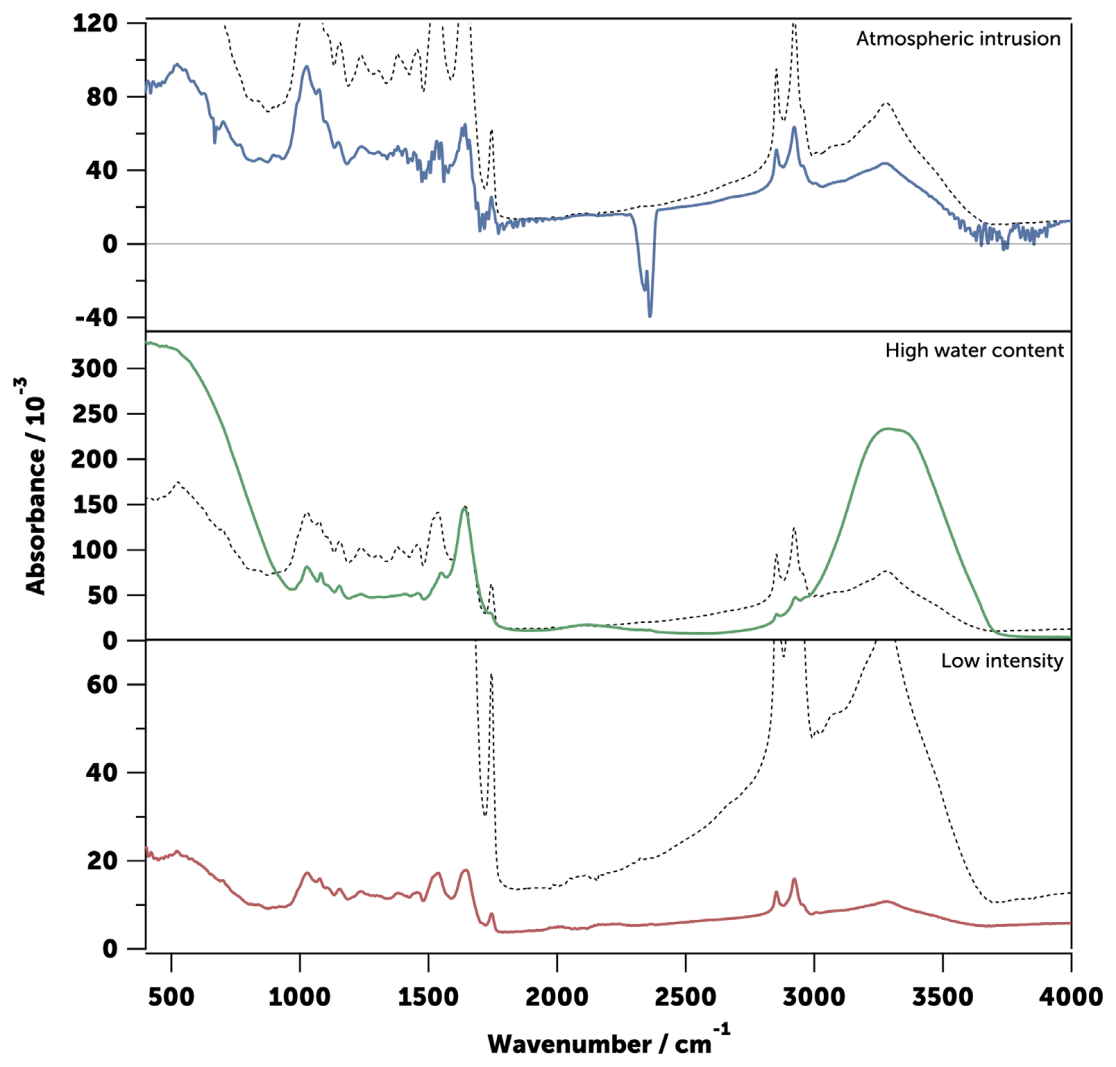
Common experimental errors during the measurement of the infrared spectrum of a mosquito using ATR-FTIR spectroscopy. Above, blue: Spectrum with a significant atmospheric intrusion. Centre, green: *An. gambiae* mosquito with high water content. Below, red. Spectrum with poorly defined features due to low intensity, caused by the displacement of the mosquito during the measurement. All spectra are compared to a correct spectrum of a mosquito shown with a black dashed line.

Dried specimens were laid on their sides on the ATR diamond so that the surface of the diamond was mainly covered by the insect’s head and thorax to avoid as far as possible measuring the contents of the abdomen ( Figure 1). The wings and limbs were not removed and were used to help position the mosquito. Pressure was then applied by the anvil of the ATR and the spectrum was measured using a dry-air purged Bruker Vertex 70 spectrometer (Bruker Corporation, Billerica, Massachusetts, USA) equipped with a Globar lamp, a Deuterated Lanthanum α Alanine doped Tri-Glicine Sulphate (DLaTGS) detector, a potassium bromide (KBr) beamsplitter, and a diamond ATR accessory (Bruker Platinum ATR Unit A225). Final, noiseless spectra were produced after averaging 16 scans taken at room temperature between 400 and 4,000 cm-1 with 1 cm ^-1^ resolution. Mosquito spectra with low intensity or a significative atmospheric intrusion ( Figure 2) were discarded automatically using *Loco Mosquito* 5.0, a custom program written in Python 3.6 (see Software availability section). This program discarded unsuitable spectra by measuring the average absorbance of the plateau in the mosquito spectra between 400 and 500 cm ^-1^ and the smoothness of the region between 3,500 and 3,900 cm ^-1^ (to detect water and CO _2_ spectra intrusion).

### Machine-learning analysis

A supervised machine-learning approach was used to map the pre-selected 17 wavenumbers (see Spectroscopic Method subsection in Results) to mosquito species (either *An. gambiae* or *An. arabiensis*) and to mosquito age. In both cases, a classification approach was used. The age classes selected were mosquito ages 1, 3, 5, 7, 9, 11, and 15 days, which allowed acceptable per-age accuracy while improving on current binary cut-off of 4 days based on oviposition (and assuming no pre-gravid behaviour). These age classes were chosen as a compromise between granularity of the predictions and model performance.

**Figure 3.**
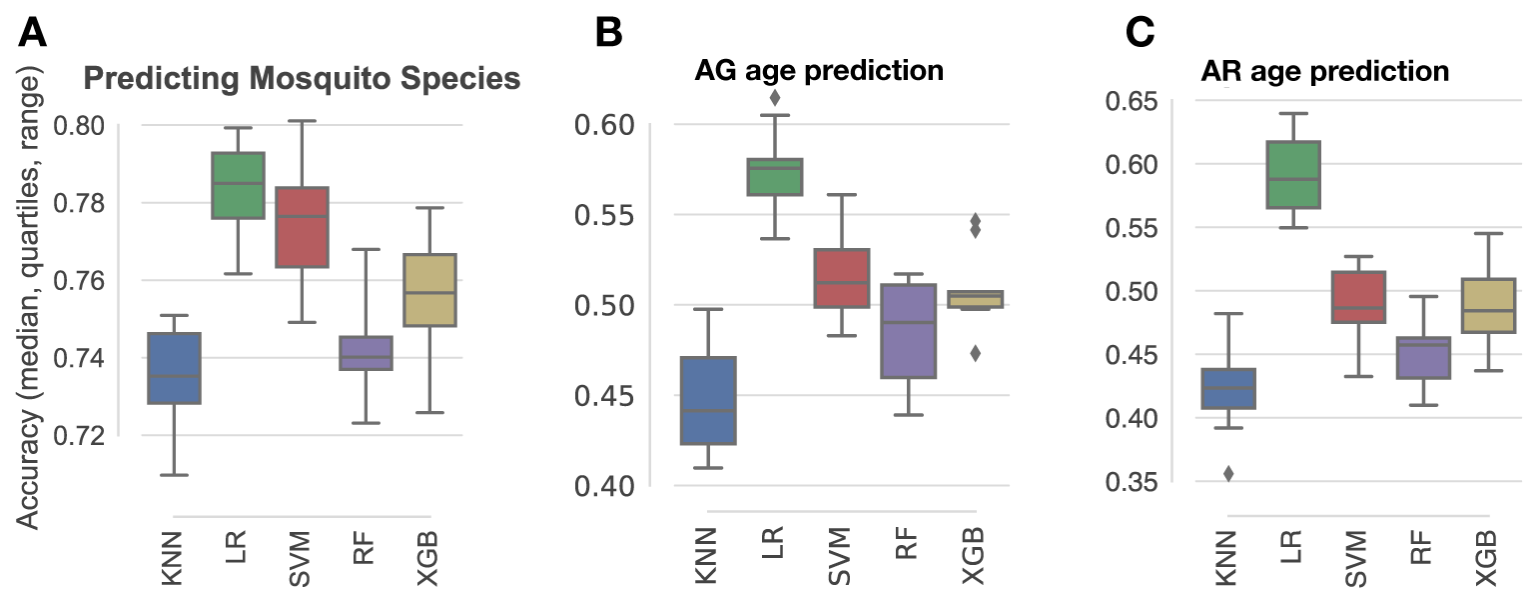
Comparison of the baseline pre-optimisation performance of 5 supervised machine learning algorithms for the prediction of mosquito species ( **A**), *An. gambiae* age ( **B**), and *An. arabiensis* age ( **C**). Each classifier was run with 5-fold cross-validation on a training subset sampled random representing stratified 70% of the full dataset and tested against the remaining 30%. No model parameter optimization was performed at this stage (please see selected models post-optimisation in Figure 13, Figure 14, and Figure 16). KNN, k Nearest Neighbours; LR, logistic regression; SVM, support vector machines; RF, random forests; XGB, gradient boosted trees with XGBoost.

Mosquito species and ages were treated in separate models to increase accuracy. To identify the algorithms most suited to the identification of either mosquito species and age class, we first compared the baseline performance of k nearest neighbours (kNN), logistic regression (LR), support vector machines (SVM), random forests (RF), and gradient boosted trees (XGB) using 5-fold cross-validation ( Figure 3). This range of parametric (LR, SVM) and non-parametric (kNN, RF, XGB) models offer different data representation schemes using Euclidian distance (kNN), linear relationships (LR, SVM), and ensemble decision trees (RF, XGB). For species and age class identification, XGB and LR, respectively, were then selected for further optimization. The full dataset—comprising 2,536 mosquito spectral features (details in Table 1) and their corresponding species or age labels—was sampled at random to generate a hold-out validation set stratified according to predicted age classes for each species (see below). The remaining samples were then repeatedly (10 rounds) split in random stratified training and test sets (10 folds). Model optimization involved a further 70%/30% random stratified splitting scheme on each of the training folds, and algorithms were trained with a broad range of parameter combinations, and the best settings for each train set retained.

**Table 1.** Number of mosquitoes of each species and status that have been measured.

*Anopheles arabiensis*																		
																		**Totals**
**Age/days**	**1**		**3**		**5**		**7**		**9**		**11**		**12**		**13**	**15**	**17**	
**Gravid**	0		57		61		41		66		70		52		90	33	80	550
**Sugar-fed**	42		43		65		67		84		67		0		39	41	16	464
**Totals**	42		100		126		108		150		137		52		129	74	96	1014
*Anopheles gambiae*																		
																		**Totals**
**Age/days**	**1**	**2**	**3**	**4**	**5**	**6**	**7**	**8**	**9**	**10**	**11**	**12**	**13**	**14**	**15**	**16**	**17**	
**Gravid**	0	0	0	47	45	51	43	40	37	39	35	89	34	0	45	61	39	605
**Sugar-fed**	160	63	65	62	54	52	59	53	53	44	41	32	27	44	44	24	40	917
**Totals**	160	63	65	109	99	103	102	93	90	83	76	121	61	44	99	85	79	1522

Each optimised model’s accuracy was then calculated against the corresponding test set. The 100 resulting trained models were then ranked according to their accuracy scores, and the best 10 retained and predictions bagged for evaluation of their predicted labels (age or species) against the true labels. All machine learning was performed in Python 3.6 using scikit-learn 0.19, XGBoost 0.82, and corresponding plotting using seaborn 0.9.

### Age-structure modelling

To illustrate the utility of our approach for field-based surveys of *Anopheles* populations, and to assess whether they could be used to measure the impact of vector control interventions in the field, we simulated age structures of *An. gambiae* and *An. arabiensis* using a simple age structure population model. Here, age corresponds to days. Specifically, the number of mosquitoes *N* surviving to from age *t* to *t*+1 was modelled as a binomial function: *N _t+_*_1_ ~ binomial ( *N _t_*, *s*); where *N _t_* is the total number of mosquitoes alive at age *t*+1 and *s* is the probability of daily survival. The daily survival rate was based on literature values, i.e., for *An. gambiae s* = 0.91 ^58^ and for *An. arabiensis* s = 0.82 ^16^. For the age structure of the populations under a theoretical intervention regime, we assume that the intervention quadruples the mortality rate of both species from day 3 onwards. This emulates a scenario where mosquitoes encounter an insecticide-treated bednet for the first time at day 3, when they start feeding.

Each age class was generated by sampling the full dataset in the proportions calculated from the above simulated age-structured populations. A continuous probability distribution was then fitted to the true and predicted discrete age distributions to better generalize our discrete model predictions to an exponentially decreasing age structure using a half-logistic probability function as

The half-logistic distribution is well-suited for fitting survival data ^59, 60^. Age distributions were compared using the Kolmogorov-Smirnov statistic on 2 samples, a two-sided test for the null hypothesis that 2 independent samples are drawn from the same continuous distribution.

### Estimation of the light penetration distance in a mosquito

The depth of light penetration for ATR measurements de-pends on the wavelength *λ* and angle of incidence of light *θ*, and on the refractive indices of the mosquito, *n*_2_, and the ATR crystal, *n*_1_:

$$d=\frac{\lambda n_{1}}{2\pi \sqrt{{\text{sin}}^{2}\theta -{\left(n_{2}-n_{1}\right)}^{2}}}$$

**Figure 4.**
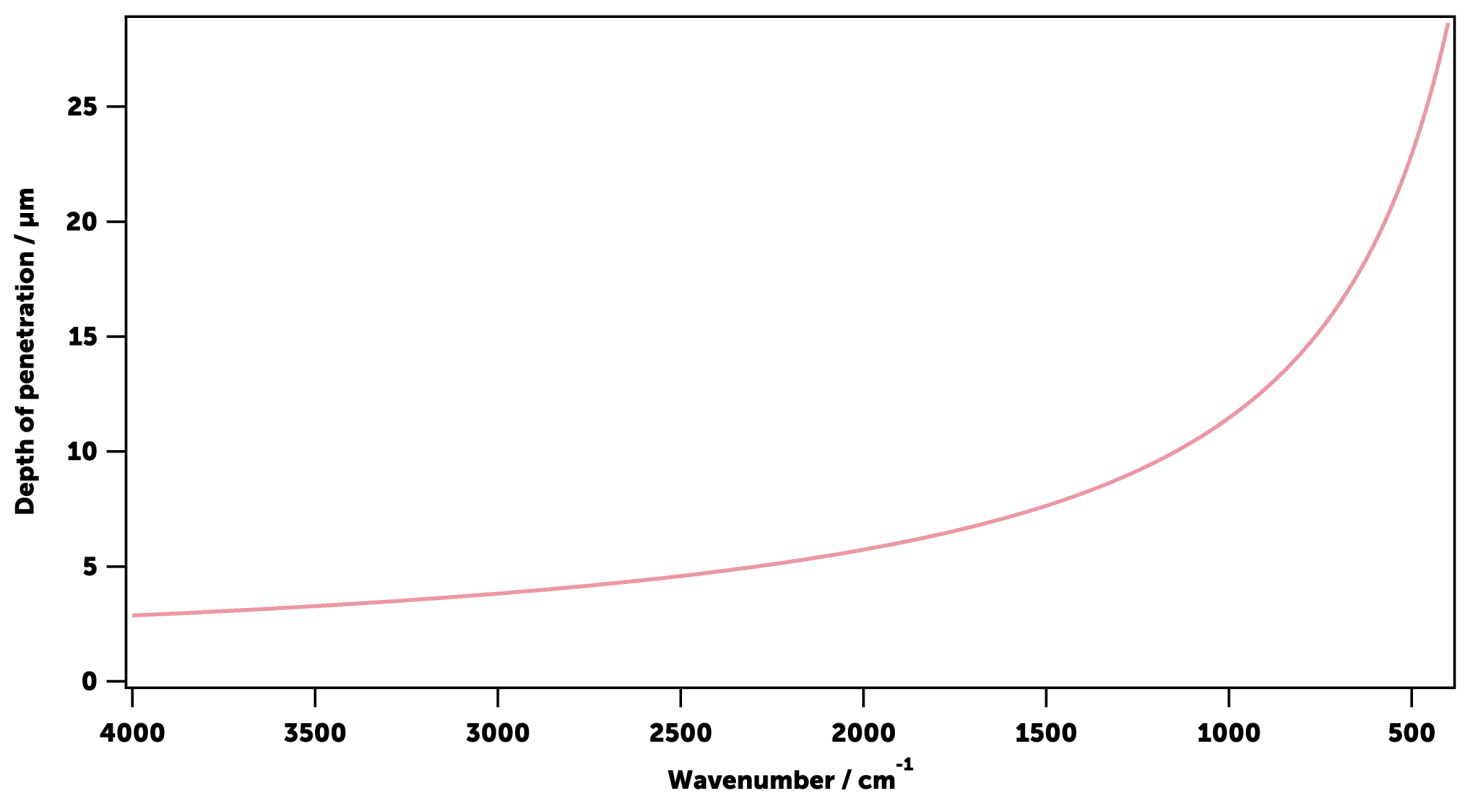
Estimated depth of penetration of the ATR evanescent wave in the mosquito sample.

Taking into account that, according to the specifications of the ATR accessory ^61^, the incidence angle is *θ* = 45º, the Sellmeier equation ^62^ for diamond ^63^ ( *λ* in μm):

$$n^{2}-1=\frac{4.3356\lambda^{2}}{\lambda^{2}-0.1060^{2}}+\frac{0.3306\lambda^{2}}{\lambda^{2}-0.1750^{2}}$$

and a Cauchy equation n( *λ*) = A + B/ *λ* ^2^, with A = 1.517 and B = 8.80·10 ^-3^ μm ^2^ for insect chitin ^64^. The results for the MIR region are shown in Figure 4.

## Results

### Mosquitoes preparation

**Figure 5.**
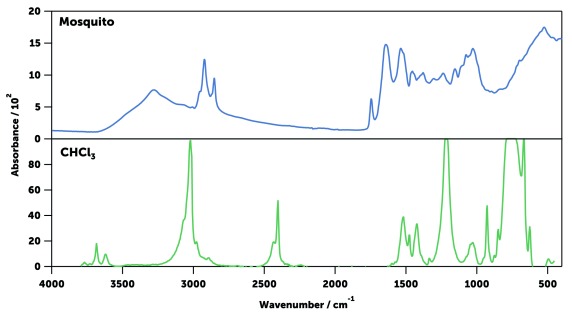
Mid-infrared absorption spectra of a typical mosquito ( *An. gambiae*, gravid, 9 days old, top) and liquid chloroform (bottom). Note the absence of the signal of the chloroform employed to kill the mosquito in the insect spectrum, since chloroform rapidly evaporates from the sample and leave no MIR-detectable signals.

**Figure 6.**
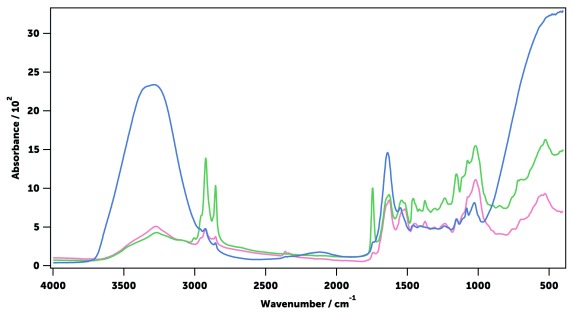
Mid-infrared absorption spectra of a recently killed mosquito (blue), a mosquito dried in a vial with silica (green) and in an oven at 80°C for 60 minutes (pink). All mosquitoes were *An. gambiae*, sugar-fed and 11 days old. A clear loss of detail can be observed in the oven dried sample due to heating.

**Figure 7.**
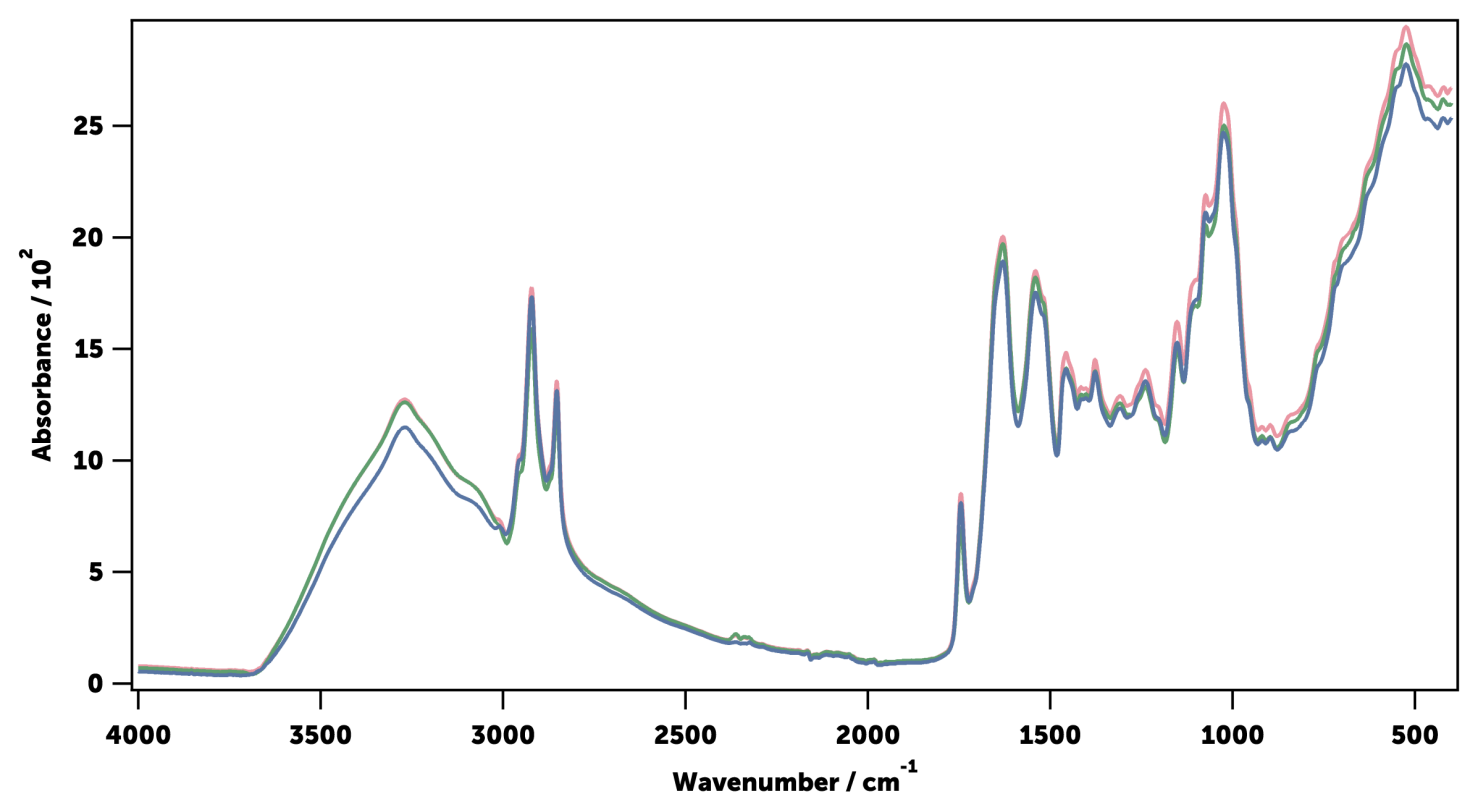
Effect of the storage time on the averaged mid-infrared spectra of 30 sugar-fed 17-day-old An. gambiae mosquitoes. 3 days (blue), 6 days (green), and 11 days (pink) after collection.

A ‘field-friendly’ protocol to kill and store mosquitoes for infrared (IR) spectroscopy was established. In brief, laboratory-reared female *An. gambiae* and *An. arabiensis* mosquitoes of different ages and physiological states were killed by exposure to chloroform for 30 minutes. As chloroform evaporates and does not interact with the mosquito cuticle, the IR spectra were not affected by this chemical ( Figure 5). This method, also used before ^40, 43, 45^, is more practical in the field than killing mosquitoes with CO _2_ or by freezing them at -20°C. Dead mosquitoes were then stored in 20 ml transport tubes with silica gel to dry them out ^65^. Removal of water from samples is essential, as it uncovers parts of the IR spectrum that would otherwise be hidden by the intense IR absorption of water ( Figure 6). Water IR absorption bands disappeared from *An. gambiae* and *An. arabiensis* mosquitoes after storage with silica gel at 4°C for one and two days, respectively (longer in a *An. arabiensis* due to its higher body water content) ^66^. In addition, this drying method preserved mosquitoes from decomposition for more than 10 days ( Figure 7). Alternative drying methods such as desiccating specimens in an oven at 80°C were shown to affect IR spectra, disrupting specially the peaks associated with lipids ( Figure 6), and therefore not used.

### Spectroscopic method

**Figure 8.**
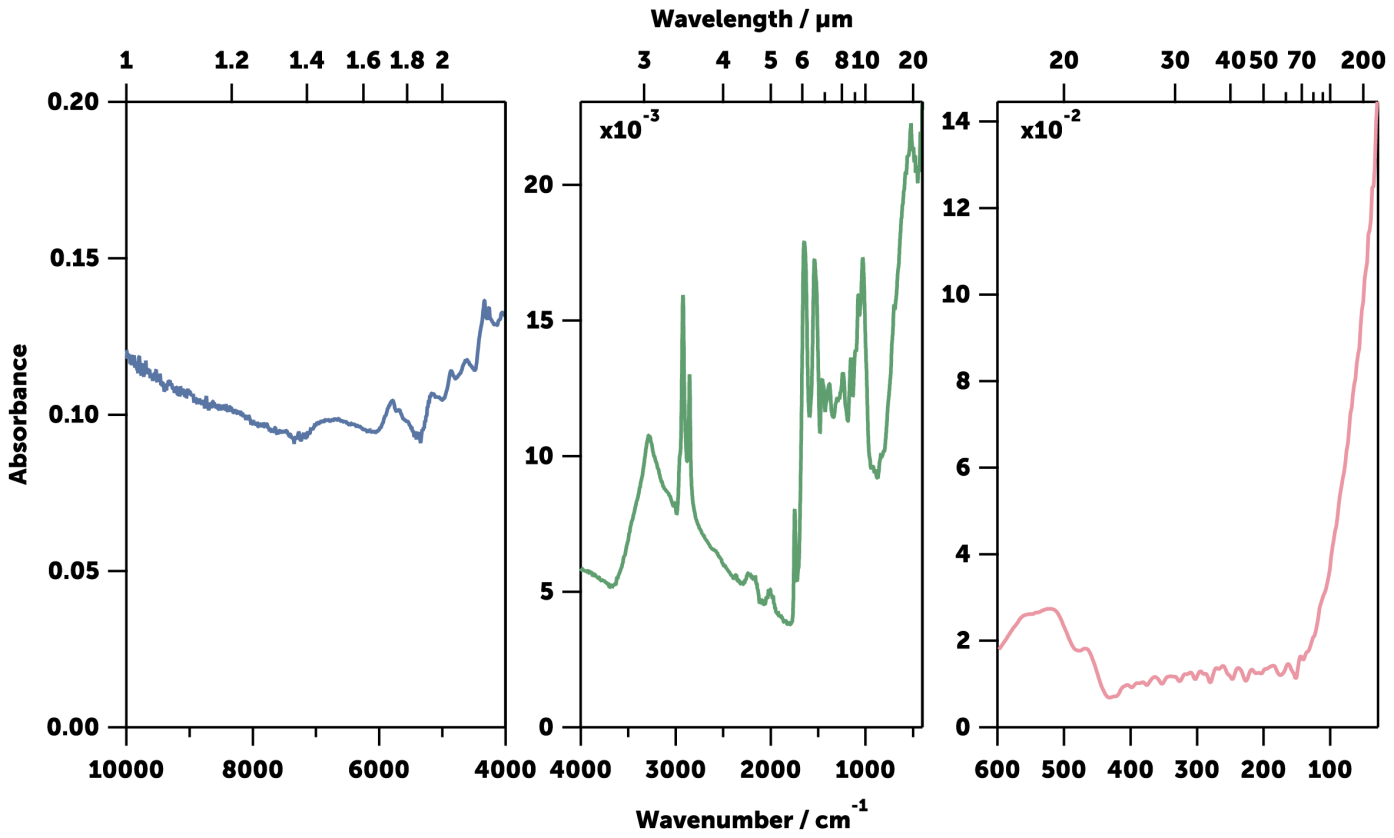
Typical near- (left, blue), mid- (centre, green), and far-infrared (right, pink) spectra of an *An. gambiae* mosquito. The near-infrared spectrum was collected using diffuse reflectance infrared spectroscopy, while the mid- and far-infrared spectra were obtained using ATR.

**Figure 9.**
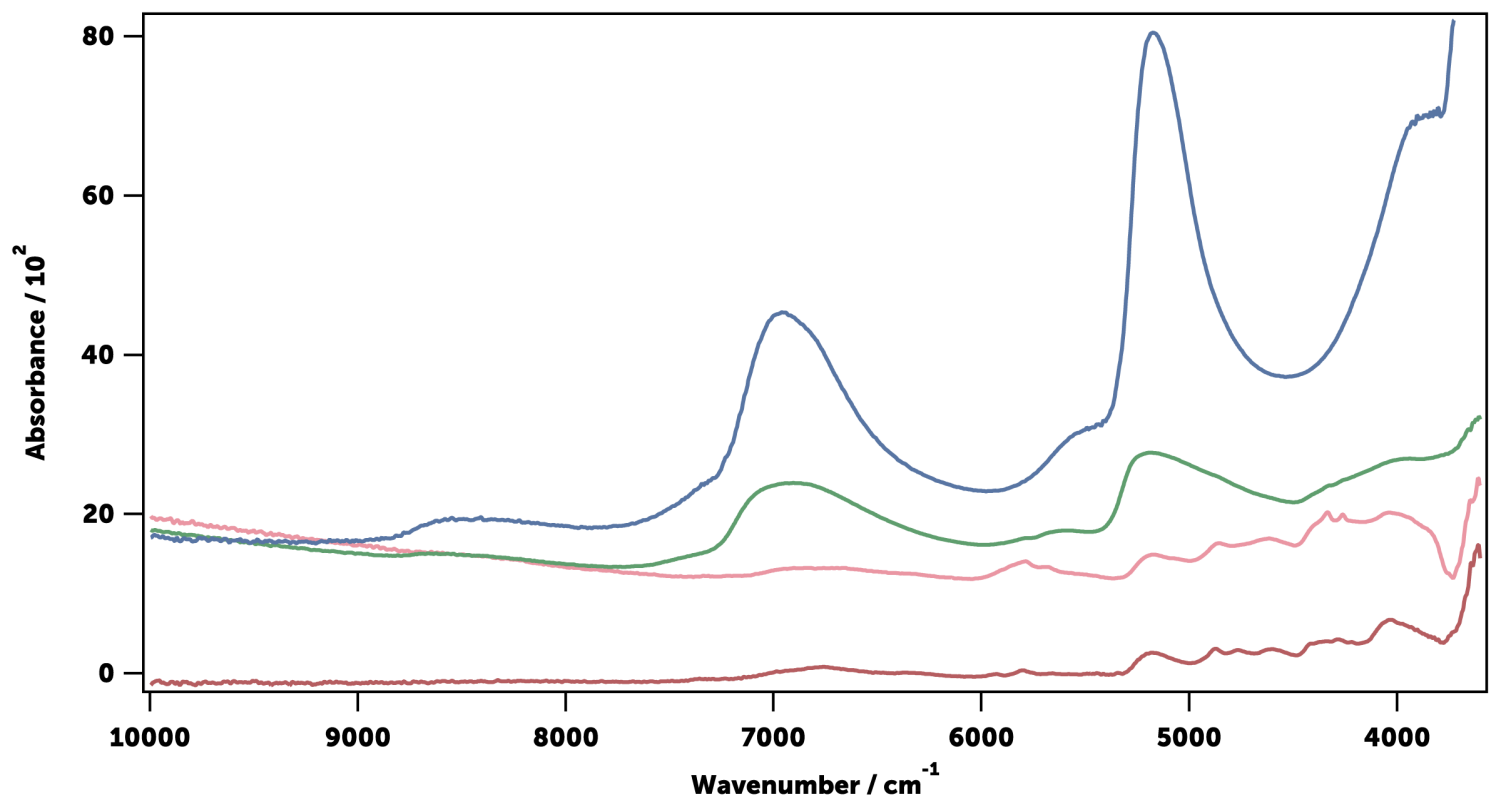
Near-infrared diffuse reflectance spectra of water (blue), an undried *An. gambiae* mosquito (green), a dried mosquito (pink), and chitin (red).

**Figure 10.**
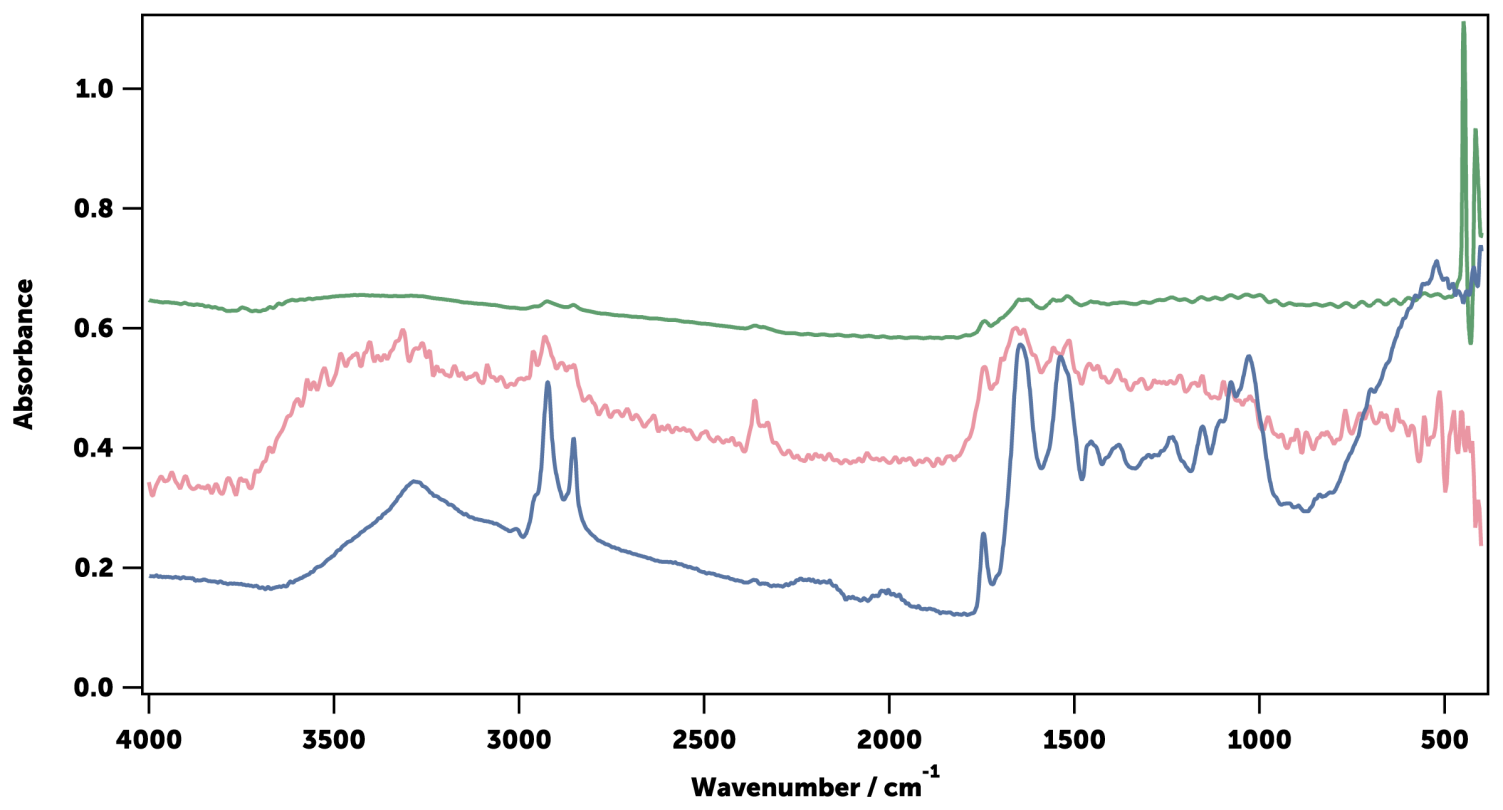
Typical ATR (blue, scaled Abs x32), diffuse reflectance (pink), and transmission (green) mid-infrared spectra of a mosquito. The transmission spectrum was taken using ZnSe windows. Its vertical offset is due to the reflection of a part of the light because of the difficulty in controlling the angle of the cell windows with the mosquito inside. The ATR, diffuse reflectance, and transmission spectra are the result of an average of 16, 120, and 80 scans, respectively.

**Figure 11.**
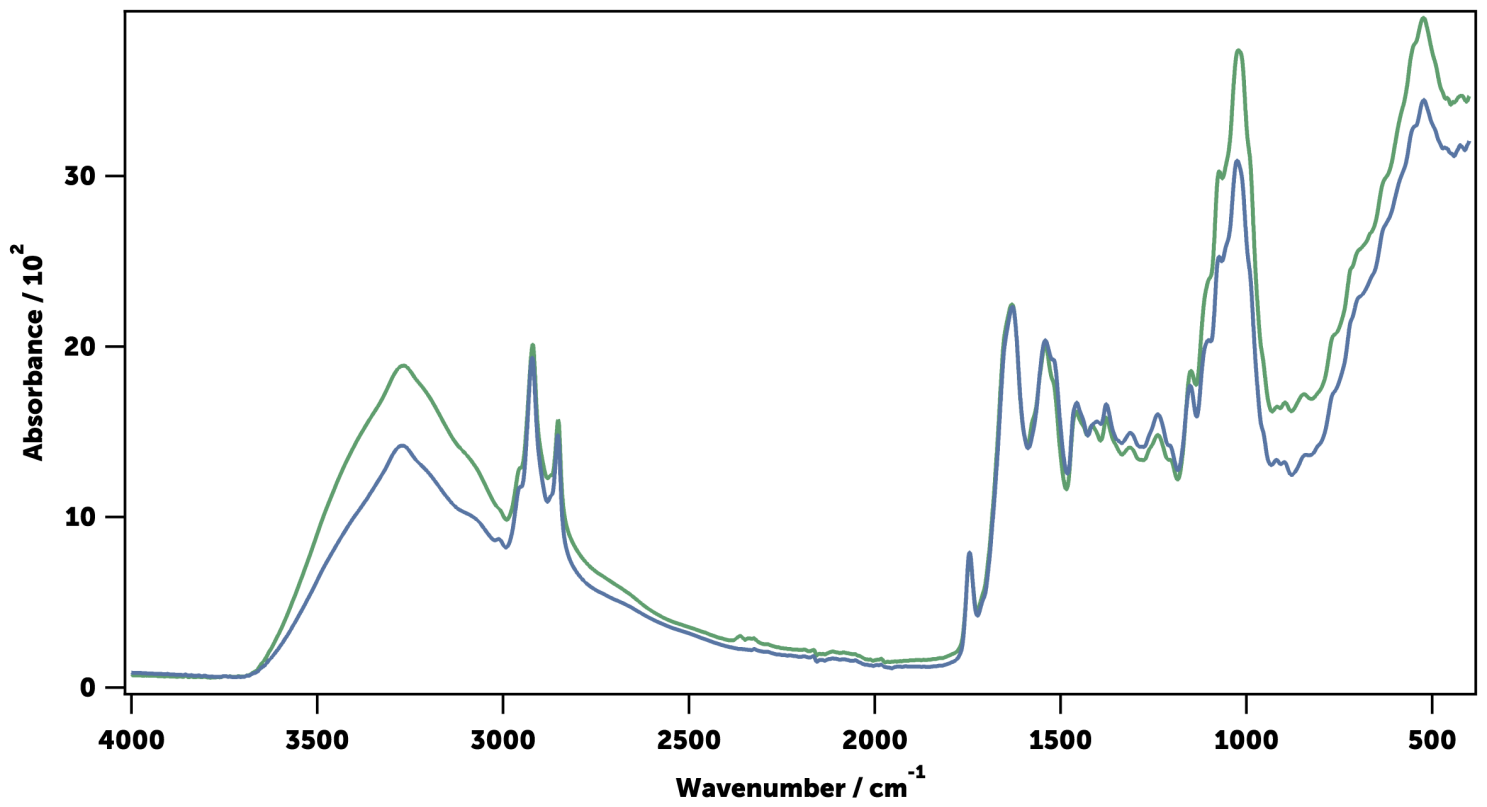
Mid-infrared absorption spectra of the head and thorax (blue) and abdomen (green) of a sugar-fed, 17-day-old, An. gambiae mosquito.

The far- (30–400 cm ^-1^), mid- (400–4,000 cm ^-1^), and near-infrared (4,000–10,000) regions of mosquito spectra were compared ( Figure 8). The far- and near-infrared regions were essentially featureless in dried mosquitoes, unlike the NIR spectra previously published ^40, 43, 45, 47, 67^ which show the intense signals of liquid water when specimens were not dried ( Figure 9). However, the mid-infrared region showed a large number of well-defined intense peaks, which are easily identifiable as coming from the chemical components of the cuticle ( Table 2). Three different IR spectral sampling techniques were investigated: diffuse reflectance, transmission, and attenuated total internal reflection (ATR, see Spectral data acquisition in Methods). ATR spectroscopy produced the best-defined and most reproducible spectra in the mid-IR region ( Figure 10). ATR also allowed the measurement of different parts of the mosquito body ( *e.g.*, head or abdomen) that have slightly different IR spectra ( Figure 11). It also had superior signal-to-noise ratios allowing acquisition of the spectra in 45 seconds. Raw spectra data is available as Underlying data ^68^.

**Table 2.** Assignment of the selected wavenumbers shown in Figure 12 ^51, 69^.

Wavenumber	Bond	Reference
3856	*	
3400	O-H	Mosquito moisture
3276	N-H	Chitin, proteins
2923	C-H _2_	Proteins, waxes
2859	C-H _2_	Proteins, waxes
1901	*	
1746	C=O	Proteins, waxes
1636	C=O	Proteins, chitin
1539	O=C-N	Proteins, chitin
1457	C-CH _3_	Wax, proteins
1307	C-N	Proteins, chitin
1154	C-O-C	Chitin, waxes
1076	C-O	Chitin
1027	C-O	Chitin
880	*	
526	C-C	Proteins, chitin
401	*	

* Wavenumbers selected as indicators of overall spectra intensity and offset.

**Figure 12.**
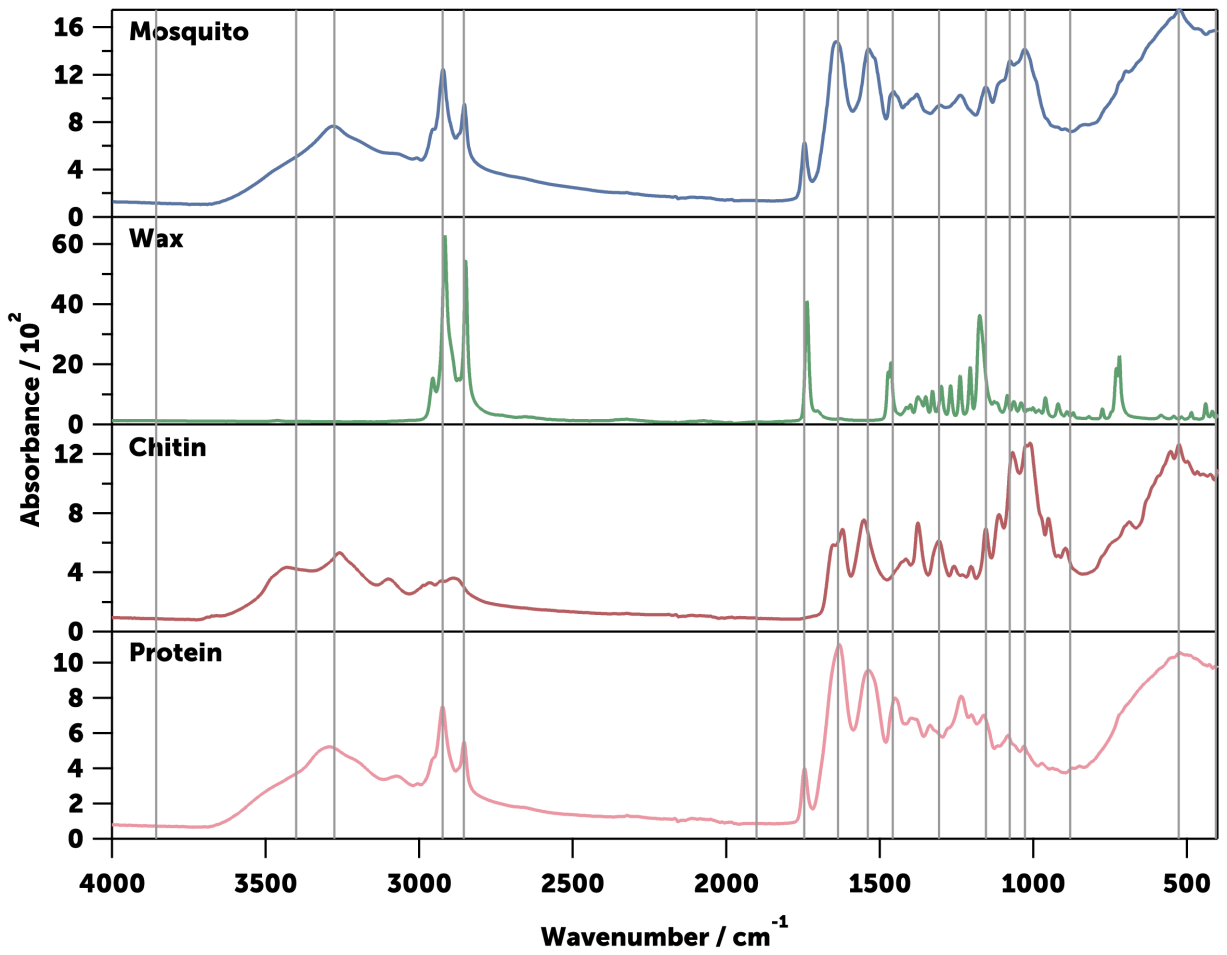
Typical mid-infrared spectrum of an Anopheles mosquito. Shown are *An. gambiae* (gravid, 9 days-old, blue) and its main chemical constituents wax (arachidyl dodecanoate, green), chitin (from shrimp shells, red), and protein (collagen from bovine Achilles tendon, pink). The wavenumbers selected for the machine learning are indicated with a grey line ( Table 2).

It was estimated that by using the ATR sampling technique in the mid-IR, the light penetrates the sample by 3–10 μm up to about 1000 cm ^-1^, and then up to 22 μm within 1000 and 400 cm ^-1^ (see Estimation of the light penetration distance in a mosquito in Methods). As the cuticle of a mosquito is approximately 2–5 μm thick ^22, 70^, the measured spectra encompass the outer shell and part of the interior of insects. As the cuticle is mainly composed of chitin, proteins, and lipids, spectra associated with these substances were individually compared with the whole-mosquito spectra ( Figure 12) to allow the assignment of the main vibrational modes of the mosquito cuticular constituents to each element ( Table 2). As the cuticular chemical composition is known to change with species and age ^71, 72^, so too are the relative magnitudes of these vibrational bands. To quantify this change, 17 wavenumbers in the MIR spectrum were selected corresponding to 13 well-defined vibrational absorption peaks (contributed in different proportions by the three main constituents) and 4 troughs (that provide information on spectrum intensity and offset). These 17 wavenumbers were then used for training machine learning models (see below).

### Mosquito species determination

To develop a MIRS-based method to determine the age and species of *An. gambiae* and *An. arabiensis*, mosquitoes were reared under laboratory conditions (see Mosquito rearing, blood feeding, and processing in Methods) and collected at ages ranging from 1 to 17 days. To model part of the variability typical in the wild, female encompassing a range of physiological states were incorporated in analysis including those that have just taken a blood meal (blood fed), those that had eggs developed in the abdomen (gravid), or that laid eggs but have not blood-fed yet again (sugar fed); mosquitoes undergone either single or multiple gonotrophic cycles depending on their age. In most cases, over 40 mosquitoes per age and physiological condition from each species were analysed ( Table 1).

**Figure 13.**
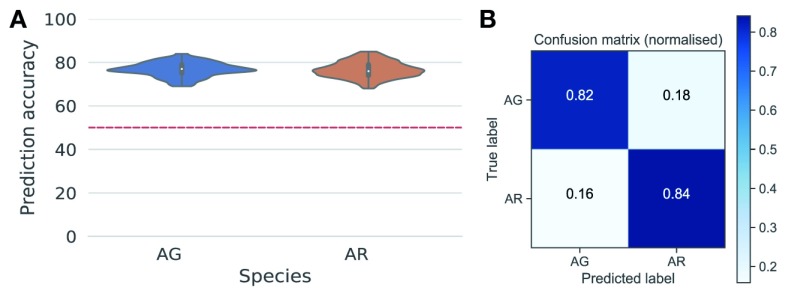
Prediction of mosquito species using mid-infrared spectra. ( **A**) Violin plots of the distribution of per species prediction accuracies of 100 models trained on different random stratified subsets (70/30 splits) of the data. The red line shows model prediction accuracy under chance alone (i.e. in the absence of learning). ( **B**) Confusion matrix showing the proportion of accurate (diagonal) classification of mosquitoes as either *An. gambiae* (AG) or *An. arabiensis* (AR) using the 10 best logistic regression models (n = 2,536).

A total of 1,522 *An. gambiae* and 1,014 *An. arabiensis* spectra from different ages and physiological conditions were used to train supervised machine-learning models (see Machine-learning analysis in Methods). Five algorithms were tested on the dataset to predict mosquito species ( Figure 3A). This initial approach identified logistic regression (LR) as the most accurate approach. We generated 100 bootstrapped models trained on and tested against different subsets of the data which, when aggregated (bagged), predicted the species identity of *An. gambiae* and *An. arabiensis* with 76.8 and 76.6% accuracy, respectively ( Figure 13 A). To increase the accuracy of the prediction while retaining the stability and generalisability afforded by bagging, we selected the 10 best models among them, which achieved 82.6% accuracy ( Figure 13 B). These results demonstrate that the MIRS signal is indicative of mosquito species and can be used to distinguish between species in a more time and cost-efficient method, although currently with less accuracy, than standard PCR methods.

### Mosquito age determination

**Figure 14.**
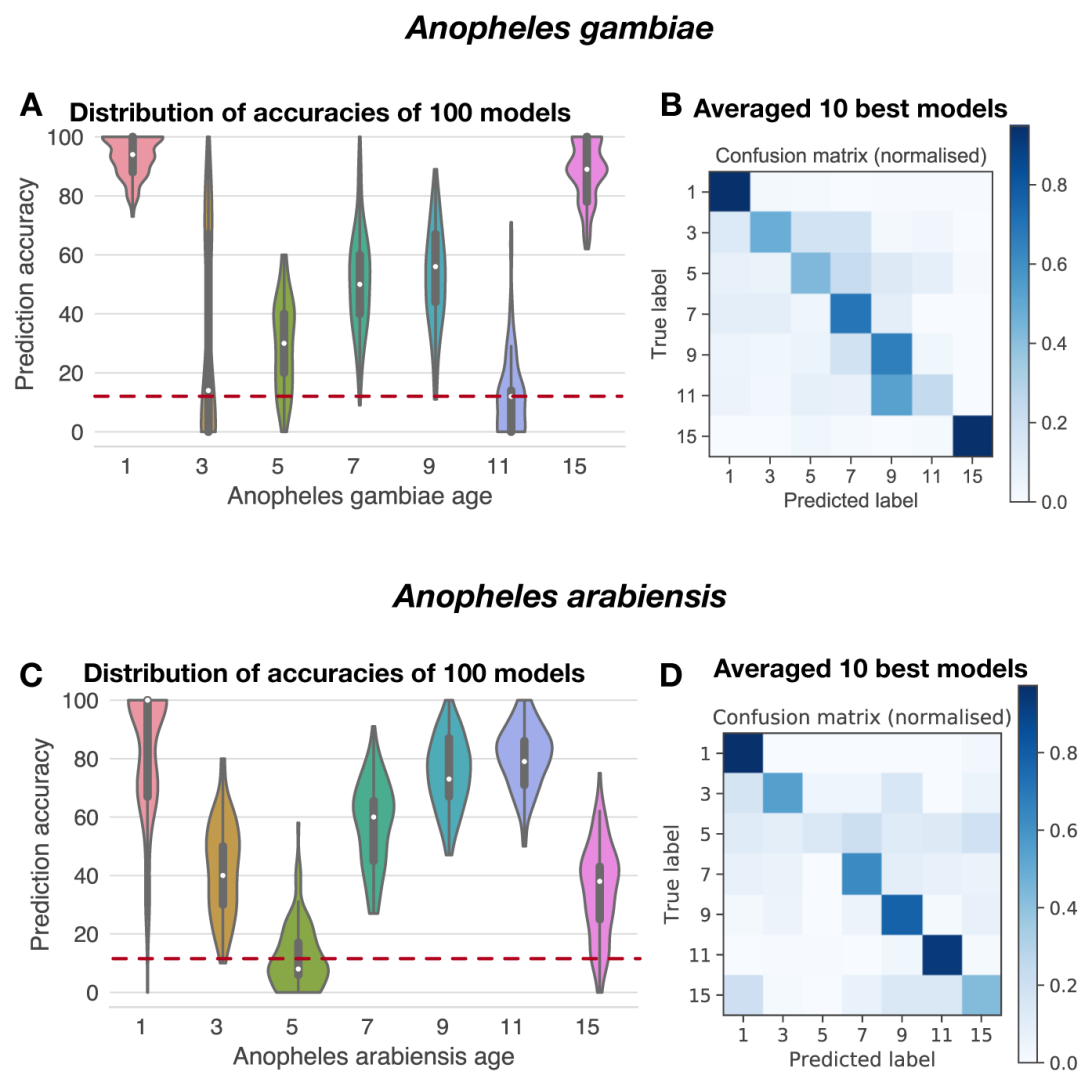
Prediction of *An. gambiae* ( **A**– **B**) and *An. arabiensis* ( **C**– **D**) age class using mid-infrared spectra. ( **A**, **C**) Violin plots of the distribution of per age class prediction accuracies of 100 optimised models. ( **B**, **D**) Confusion matrices showing the proportion of accurate (diagonal) classification of mosquitoes as either 1, 3, 5, 7, 9, 11, or 15 days old using the 10 best logistic regression models trained on repeated stratified random subsets using 70% of all mosquitoes sampled, and tested on the remaining 30% (n = 681 for *An. gambiae* and n = 737 for *An. arabiensis*).

**Figure 15.**
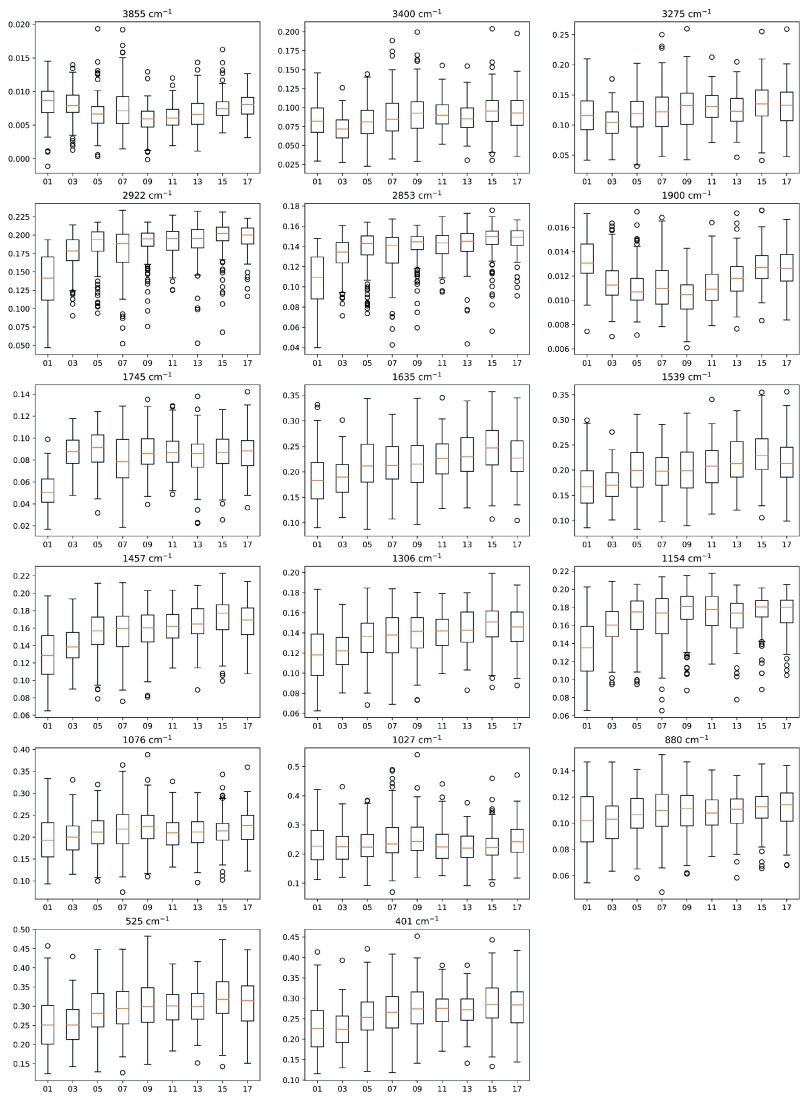
Box-whisker plot containing the measured absorption in each wavenumber and for each age for all the mosquitoes ( *An. arabiensis* and *An. gambiae*). The orange lines represent the median absorbance of each age and wavenumber, the limits of the boxes correspond to the interquartile range (IQR) and the whiskers show the lowest datum still within 1.5 IQR of the lower quartile, and the highest datum still within 1.5 IQR of the upper quartile.

After the development of the species-prediction model, a similar supervised machine-learning approach was used to model the chronological age for a given mosquito species. Mosquitoes were screened every second day after emerging as adults, and models trained on the same set of 17 wavenumbers as above. The LR model again performed best for both species in correctly mapping wavenumber intensities to mosquito age ( Figure 3B and C). To train, optimise, and validate the models, the full dataset was partitioned into an age-structured validation set and retained for later use in population models (see below). The remaining samples were then randomly split into stratified 70%/30% training and test sets for model tuning. The accuracy in predicting each chronological age varied over mosquito lifespan and between species, ranging from an average of 15% to 97% for *An. gambiae* and 10% to 100% for *An. arabiensis* ( Figure 14). As in previous studies ^40, 43^, it was found that the chronological age of young and old mosquitoes was generally more accurately predicted than intermediate ages, although there were some differences between species. These results suggest that the MIRS-based approach developed here can predict the chronological age of each species from 1 to 15 days old, as well as providing the confidence of prediction for each age class. Furthermore, a trade-off was observed between the granularity of the prediction and its accuracy: models trained on daily scans (not shown) performed worse than if we allowed the mosquitoes to age 2 or 3 days between each scan, suggesting that the ageing of the mosquito cuticle varies between individuals and that the features used for training the models overlap between consecutive age classes ( Figure 15).

### Predicting mosquito age structure

To monitor the efficacy of vector control interventions in the field, accurately describing the age distribution ( *i.e.*, the summary demographic age structure of the local vector species population) is more important than knowing the age of any individual mosquito ^46^. Consequently, we tested how well the above age prediction models developed for *An. gambiae* and *An. arabiensis* could reconstruct the known age distribution of mosquito populations. Mosquito populations reflecting anticipated changes in mortality were used under two scenarios: natural mortality and increased mortality due to a theoretical vector control interventions.

Consistent with natural mosquito populations, but unlike our training dataset, field sampling would not produce age-balanced sample sizes, but rather diminishing sample sizes at older age classes. Furthermore, it would be highly desirable to use our models to measure the impact of vector control interventions on mosquito-population age structures. However, because no real datasets of a true mosquito population age structure exist, the age structures of *An. gambiae* and *An. arabiensis* were modelled based on their reported average daily mortality ^16, 58^ and assuming an intervention that increased the mortality of adult females four-fold after a first blood meal (~3 days after adult emergence).

**Figure 16.**
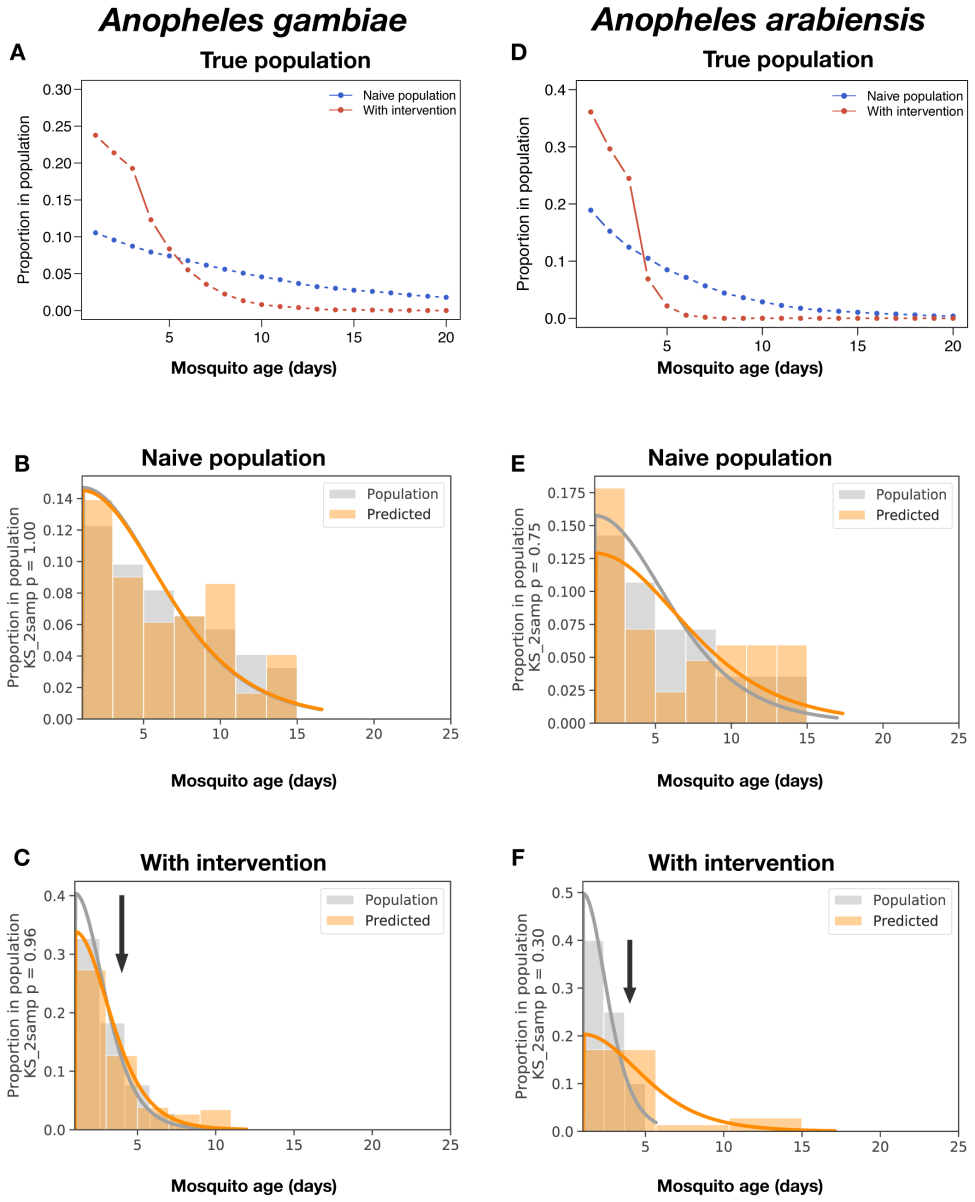
Reconstruction of the age structure of simulated populations of *An. gambiae* and *An. arabiensis* mosquitoes sampled from simulated pre- and post-treatment populations. Population age structures of *An. gambiae* ( **A**– **C**) and *An. arabiensis* ( **D**– **F**) were generated using an age structure population model assuming survival rates of 0.91 ( *An. gambiae*, **A**) or 0.82 ( *An. arabiensis*, **D**), under two common scenarios: naive untreated populations (blue lines), and populations in which a simulated vector control program resulted in 4x daily mortality of mosquitoes after day 3 (see Age-structure modelling in Methods for details). The proportions of each age class were extracted from those simulated populations ( **A**, **D**), and used to build datasets that are representative of a field-sampled population survey (grey bars in **B**, **C**, **E**, and **F**). The resulting age-structured dataset was then used as the test set for our age-predicting machine learning models (see Figure 3) and compared with the predicted age structure generated from those models (orange bars in **B**, **C**, **E**, and **F**). Finally, we fit a continuous probability distribution to the true (grey curve) and predicted (orange curve) for better generalization of our discrete model predictions to an exponentially decreasing age structure. Population distributions were compared using a 2-sample Kolmogorov-Smirnov test (KS_2samp), reported in the y-axis labels. A - Relative proportion of each age class in a simulated population of *An. gambiae*. B - Estimation of age structure of simulated population from ( **A**) using best models from Figure 3B for *An. gambiae* (n = 130). C - Estimation of age structure of simulated population post-intervention from ( **A**) using best models from Figure 3B for *An. gambiae* (n = 122). D - Relative proportion of each age class in a simulated population of *An. arabiensis*. E - Estimation of age structure of simulated population from ( **A**) using best models from Figure 3D for *An. arabiensis* (n = 42). F - Estimation of age structure of simulated population post-intervention from ( **A**) using best models from Figure 3D for *An. arabiensis* (n = 45).

In these simulations, a starting population of 1,000 female mosquitoes was used, with the population at each subsequent day being calculated as a proportion of the previous day, with survival rates for each species estimated from reports on field studies ( Figure 16A,D) ^16, 58^. The resulting age-structured populations were then used to randomly sample replicates in the corresponding proportion for each age class used in our MIRS-based prediction models ( Figure 15B–F, grey bars; n = 122 for *An. gambiae* and n = 42 for *An. arabiensis*). The models trained above were then used to predict age classes from the MIRS of this age-structured population ( Figure 16B–F, orange bars).

To test the ability of those models to reconstruct the age structure of the true population from our predicted age class frequencies, the age structures of the predicted ( Figure 16B–F, orange bars) and true sampled populations ( Figure 16B–F, grey bars) were modelled with the best fit half-logistic distribution for each species (grey and orange curves in Figures 15 B,C and E,F; see also Age-structure modelling in Methods). The true and predicted age distributions were statistically indistinguishable (Kolmogorov-Smirnov 2-sample test (KS test), p = 1 and p = 0.99 for *An. gambiae* pre- and post-intervention, respectively; p = 0.75 and p = 0.30 for *An. arabiensis* pre- and post-intervention, respectively). This approach shows that the algorithm can reconstruct the age structure with good accuracy. Furthermore, our models detected a shift in mosquito age structure consistent with the simulated impacts of the interventions (sampled from true population: KS test p < 0.0001 for *An. gambiae* and p = 0.004 for *An. arabiensis*; predicted population: p < 0.0001 for *An. gambiae* and p = 0.1 for *An. arabiensis*), suggesting that this MIRS-based approach holds promise for robust measurement and estimation of the age structure of mosquito vector populations.

## Discussion

We developed a straightforward, inexpensive, and rapid method to determine the age and species of large numbers of *An. gambiae* s.l. mosquitoes ( *An. gambiae* s.s. and *An. arabiensis*). Based on the supervised machine-learning analysis of their mid-infrared spectra, this method facilitates prediction of mosquito species distribution and survival, two crucial tasks critical to implement and assess malaria control strategies. An advantage of this approach is that in comparison to the current most widely used technique based on dissection, it can determine the whole-age distribution of a mosquito population from the day of emergence until two weeks of age. Although the accuracy of age prediction in the “mid-range” of mosquito life span was not high, by determining the age structure of a population this method could accurately estimate the proportion of mosquitoes within the older and most epidemiologically-important age classes that responsible for malaria transmission.

The use of the mid-infrared spectral region provides some advantages over techniques using near-infrared. Foremost, it is possible to independently quantify the amount of different biochemical components as their vibrational bands appear at different wavenumbers. Furthermore, the MIRS bands are more intense and have much greater definition. In contrast, the near-infrared spectrum of a mosquito is composed of few weak signals ( Figure 8) that are typically dominated by the much stronger vibrational overtone and combination bands of water ( Figure 9) ^46^, which is likely more dependent on the mosquito physiological state and environmental conditions than on other mosquito traits, such as species and age.

We have shown that the variation of MIR spectra over mosquito age can be exploited by a machine-learning algorithm to predict the chronological age, and ultimately reconstruct population age structures of two important malaria vector species under simulated conditions of changing mortality risk due to vector control. Our algorithms accurately reconstructed age structures of both *An. arabiensis* and *An. gambiae,* and also detected shifts in mosquito age structure consistent with simulated impacts of interventions. The ability of this proposed technique to predict the age structure of a population suggests that this approach could constitute an efficient tool for monitoring the efficacy of vector control interventions. Future work will include larger datasets used for training in supervised machine learning, comprising field samples with different ecological conditions. The ecological variability of field samples has limited the use of NIRS for age prediction in wild mosquito populations ^45^. While the accuracy of MIRS-based approaches may also decline when moving from laboratory-reared to field mosquitoes, we predict that this method will be more robust due to the specific information content and high signal clarity that is obtained in spectra from MIRS. Additional improvements are anticipated by increasing the size and variability of the training set on which mosquito age predictions are validated. This will also facilitate the use of alternative machine learning techniques such as neural networks ^73^ which may yield even higher accuracy and repeatability.

We have shown that MIRS can discriminate between morphologically identical *An. gambiae* s.l. species with ~83% accuracy. While the observed accuracy of MIRS species prediction is still not comparable to the PCR precision, further work including a larger training set and field samples is expected to increase the overall accuracy of this approach. In addition, the inclusion of other species of the *An. gambiae* s.l. complex will be necessary to implement this technique for field application. However, these laboratory-based results, which included mosquitoes from different ages, physiological conditions, and cohorts, suggest that despite the ecological and life-history traits variation, MIR spectra contain a species-specific signature that the machine-learning algorithm can detect. Indeed, mass-spectrometry studies have shown that different species in the *An. gambiae* s.l. complex have quantitative differences in the cuticular hydrocarbon composition of their cuticle ^72^, which will affect the MIR spectra.

The biochemical signature obtained by MIRS from the mosquito cuticle provided information on both mosquito species and age. It may therefore be possible to obtain further information on other mosquito traits that alter the cuticular composition. Recently, a new insecticide resistance mechanism has been discovered in *An. gambiae*, which relies on an increased cuticle thickness that in turn reduces insecticide uptake ^73^. While this mechanism has been detected by electron microscopy, there are no other methods to measure this new trait, which could have profound epidemiological consequences. In the future, MIRS calibrations including cuticular resistant mosquitoes may be able to identify this insecticide resistant trait. In addition, infection with the *Plasmodium* malaria parasite might be detected by MIRS. Pathogen infection is known to alter mosquito physiology and could directly or indirectly modify their cuticular composition. For example, in the dengue and Zika vector *Aedes aegypti* mosquitoes, an infrared spectroscopy method has recently been developed to detect Zika virus ^47^, the bacterial endosymbiont *Wolbachia* ^52, 67^, and malaria infection in mosquitoes ^41, 74^.

The accuracy, speed, and generalisability of the MIRS approach presented here shows that this tool holds promise for use in the evaluation of vector control interventions and as triage method when a large number of specimens (>500 -1000) requires to be processed in a rapid fashion. The inclusion of new species, larger sample sizes and field samples with variable ecological conditions is a prerequisite for the application of this technique. It is worth noting that the cost of a portable FTIR MIR spectrometer is ~$20–25,000, which is in the range of quantitative PCR machines used for species determination and/or insecticide resistance monitoring. However, in contrast to PCR analysis, no additional, ongoing costs for reagents and running costs are required once the core equipment is installed. Thus, this approach could be particularly valuable in resource limited settings.

The MIRS method presented here provides rapid and accurate information on *Anopheles* species (82.6%) and reliably characterises mosquito age distribution. However, these results were obtained by training machine learning models with a relatively modest number of mosquitoes (2,536). In future work, it will be possible to generate much larger MIRS datasets and thus train more sophisticated predictive models. Such larger data sets will lend themselves to analysis by more powerful “big data” approaches including deep learning methods that would be expected to improve accuracy considerably beyond this proof-of-principle study. Furthermore, the technique applied to malaria vectors here could also be expanded to other vector-borne diseases such as Zika, dengue, Lyme disease, leishmaniasis, or filariasis. In light of these opportunities, we recommend this method be prioritised for further evaluation.

## Data availability

### Underlying data

Enlighten: Research Data: Prediction of malaria mosquito species and population age structure using mid-infrared spectroscopy and supervised machine learning. https://doi.org/10.5525/gla.researchdata.688 ^68^

This project contains the following underlying data:

- DataMosquitoes.zip (zip file containing underlying spectra data)

## Software availability

Source code: https://github.com/SimonAB/Gonzalez-Jimenez_MIRS/tree/v1.0

Archived source code: http://doi.org/10.5281/zenodo.2609356 ^75^

Licence: GNU General Public License v3.0
