# Supplementary material for: Prediction of mosquito species and population age structure using mid-infrared spectroscopy and supervised machine learning
Source: Wellcome Open Res. 2019 Sep 16;4:76. Originally published 2019 May 1. [Version 3] doi: 10.12688/wellcomeopenres.15201.3 (PMC6753605; doi:10.12688/wellcomeopenres.15201.3)
Supplement: Peer review history

# Peer review: Reviewer response for version 3

**DOI:** 10.21956/wellcomeopenres.16923.r36486

**Published:** 2019-09-18

**Authors:** Yoosook Lee

**Affiliations:** 1 Department of Pathology, Microbiology and Immunology, University of California, Davis, Davis, CA, USA

All concerns were addressed.

I confirm that I have read this submission and believe that I have an appropriate level of expertise to confirm that it is of an acceptable scientific standard.

# Peer review: Reviewer response for version 2

**DOI:** 10.21956/wellcomeopenres.16821.r36164

**Published:** 2019-08-21

**Authors:** Yoosook Lee

**Affiliations:** 1 Department of Pathology, Microbiology and Immunology, University of California, Davis, Davis, CA, USA

Thanks for the revision.

- Please include the following method (Joy *et al*. 2012 ^1^) to be part of the introduction paragraph starting "Given these problems with ovary-based assessment, there has been significant investigation of alternative, molecular based approaches..."
- Figure 3 legend - list the items in incorrect order. Needs to be fixed.
- Figure 8 legend - please provide the wavelength numbers in the legend as in the text.
- Figure 13 - the numbers provided in the confusion matrix (e.g. 82% accuracy in AG classification) doesn't seem to be consistent with the violin plot showing the median less than 80% in both species. Given explanation of the text, it seems like the Figure 13A is still not the final model that provided 82% accuracy. Why not put the results of the best model you found here instead of intermediate result?
- Also put a unit in parenthesis (e.g. %) after prediction accuracy in Figure 13A and Figure 14A and 14C.
- The section of "Predicting mosquito age structure" is problematic. The condition of "increased mortality due to vector control interventions" is not clearly stated. What type of vector control do you have in mind? What's the basis for age-dependent mortality for this? This is also based on a model that is poorly performed in most age groups. I would cut this part and reserve for future studies with improved age prediction models and with some base field data. It is hard to evaluate at current stage.
- This is not necessary condition for indexing but I wonder if the authors would be open to investigate more coercer age groups like in Joy *et al*. 2012 ^1^ - e.g. nonvectors (0–5 days post-emergence), unlikely vectors (6–14 days post-emergence), and potential vectors (15+ days post-emergence) and see if the MIRS would perform better in predicting those age groups (the exact ranges subject to change based on the consensus of the previous reports).
- "We have shown that the variation of MIR spectra over mosquito age can be exploited by a machine-learning algorithm to predict the chronological age with a high degree of accuracy, and ultimately reconstruct population age structures of two important malaria vector species under simulated conditions of changing mortality risk due to vector control". Change to "We have shown that the variation of MIR spectra over mosquito age can be exploited by a machine-learning algorithm to predict the chronological age."
- Also change discussion consistent with the removal of modeling section. For example, change "The ability of this proposed technique to predict the age structure of a population suggests that this approach could constitute an efficient tool for monitoring the efficacy of vector control interventions." to "The model can be used to predict the age structure of field population under various vector control interventions that may impact certain age groups more than others."
- For the discussion paragraph starting "We have shown that MIRS can discriminate between morphologically identical *An. gambiae s.l. *...." I would add a sentence about how this method could be used as a triage method involving large number (>500-1000) of specimens that needs to be processed in rapid fashion.
- "...increased cuticle thickness that in turn reduces insecticide resistance uptake" - the following reference by Wood *et al*. (2010) ^2^ seems more appropriate citation for this. Also based on studies by Cook *et al*. (2006) ^3^ it seems like cuticle thickness would change over time and mature at the age of 10 or above. If true, the unreliability of age determination on the present study may result from the varying degree in speed of maturation at individual level. It may be useful to have some study to correlate cuticle thickness or kdr genotype and MIRS spectrum at different age groups to determine the maturation of any particular insecticide resistatance trait or cross interaction between kdr genotype, cuticle thickness and insecticide resistance phenotype.
- For journal team, a link to the "Supplementary Note 1" in the Results-Mosquitoes preparation section would be very helpful to navigate the content of the article.

I confirm that I have read this submission and believe that I have an appropriate level of expertise to confirm that it is of an acceptable scientific standard, however I have significant reservations, as outlined above.

## References

[1] : Aging field collected Aedes aegypti to determine their capacity for dengue transmission in the southwestern United States. *PLoS One*.2012;7(10) : 10.1371/journal.pone.0046946 e46946 10.1371/journal.pone.0046946 23077536PMC3470585

[2] : Cuticle thickening associated with pyrethroid resistance in the major malaria vector Anopheles funestus. *Parasit Vectors*.2010;3: 10.1186/1756-3305-3-67 67 10.1186/1756-3305-3-67 20684757PMC2924294

[3] : The use of transcriptional profiles to predict adult mosquito age under field conditions. *Proc Natl Acad Sci U S A*.2006;103(48) : 10.1073/pnas.0604875103 18060-5 10.1073/pnas.0604875103 17110448PMC1838706

# Response

**Published:** 2019-08-28

**Authors:** Klaas Wynne

**Affiliations:** University of Glasgow, UK

Reply to reviewer comments on “Prediction of mosquito species and population age structure using mid-infrared spectroscopy and supervised machine learning [version 2; peer review: 1 approved with reserves]”

Dear Dr. Lee:

Thank you very much for your second report. We really appreciate your valuable comments that have allowed us to improve our manuscript and remove the remaining typos. Please find below our response to your point specific comments:

- Please include the following method (Joy *et al*. 2012) to be part of the introduction paragraph starting "Given these problems with ovary-based assessment, there has been significant investigation of alternative, molecular based approaches..."

Suggestion attended to.

- Figure 3 legend - list the items in incorrect order. Needs to be fixed.

Suggestion attended to.

- Figure 8 legend - please provide the wavelength numbers in the legend as in the text.

Suggestion attended to.

- Figure 13 - the numbers provided in the confusion matrix (e.g. 82% accuracy in AG classification) doesn't seem to be consistent with the violin plot showing the median less than 80% in both species. Given explanation of the text, it seems like the Figure 13A is still not the final model that provided 82% accuracy. Why not put the results of the best model you found here instead of intermediate result?
- Also put a unit in parenthesis (e.g. %) after prediction accuracy in Figure 13A and Figure 14A and 14C.

We thank the reviewer, but respectfully disagree. The violin plots in Fig 13A describe the performance of multiple models trained on different subsets of the full dataset – they describe a population of model-dataset mappings from which we selected a ‘best model’ shown in Fig 13B. It is important to show this distribution to be as clear as possible about how the algorithms perform for different subsets of the data and to avoid any doubt about our model selection process. It is also an important figure for supporting the generalisability of our models: regardless of the composition of the training and test sets, the models tend to perform far better than random (red line in Fig 13A). We have clarified the main text of the results and the legend of Figure 13 accordingly.

- The section of "Predicting mosquito age structure" is problematic. The condition of "increased mortality due to vector control interventions" is not clearly stated. What type of vector control do you have in mind? What's the basis for age-dependent mortality for this? This is also based on a model that is poorly performed in most age groups. I would cut this part and reserve for future studies with improved age prediction models and with some base field data. It is hard to evaluate at current stage.

We believe we have already described what type of intervention we have assumed (i.e. an insecticide-treated bed net) as detailed in the method section “ *Age-structure modelling*”, where indeed this reads: “For the age structure of the populations under a potential intervention regime, we assume that the intervention quadruples the mortality rate of both species from day 3 onwards. This emulates a scenario where mosquitoes encounter an insecticide-treated bednet for the first time on day 3, when they start feeding”. To further clarify this, we have now explicitly stated this is a *theoretical* vector control intervention.

We wish to retain this part as this provides a theoretical example of how these predictions and relative modelled age structures perform under different scenarios. We agree that field data will be essential for the empirical evaluation of the technique, which is over the scope of this work, where instead we have evaluated the approach under two theoretical (but plausible) age structure scenarios.

- This is not necessary condition for indexing but I wonder if the authors would be open to investigate more coercer age groups like in Joy *et al*. 2012 - e.g. nonvectors (0–5 days post-emergence), unlikely vectors (6–14 days post-emergence), and potential vectors (15+ days post-emergence) and see if the MIRS would perform better in predicting those age groups (the exact ranges subject to change based on the consensus of the previous reports).

Thank you for this idea. We will take it into account in our next experiments.

- "We have shown that the variation of MIR spectra over mosquito age can be exploited by a machine-learning algorithm to predict the chronological age with a high degree of accuracy, and ultimately reconstruct population age structures of two important malaria vector species under simulated conditions of changing mortality risk due to vector control". Change to "We have shown that the variation of MIR spectra over mosquito age can be exploited by a machine-learning algorithm to predict the chronological age."

Suggestion attended to.

- For the discussion paragraph starting "We have shown that MIRS can discriminate between morphologically identical An. gambiae s.l. ...." I would add a sentence about how this method could be used as a triage method involving large number (>500-1000) of specimens that needs to be processed in rapid fashion.

Thank you very much for this comment. We have added a similar sentence to the text following to your suggestion.

- "...increased cuticle thickness that in turn reduces insecticide resistance uptake" - the following reference by Wood *et al*. (2010) seems more appropriate citation for this. Also based on studies by Cook *et al*. (2006) it seems like cuticle thickness would change over time and mature at the age of 10 or above. If true, the unreliability of age determination on the present study may result from the varying degree in speed of maturation at individual level. It may be useful to have some study to correlate cuticle thickness or kdr genotype and MIRS spectrum at different age groups to determine the maturation of any particular insecticide resistatance trait or cross interaction between kdr genotype, cuticle thickness and insecticide resistance phenotype.

We have changed the reference. Also, we appreciate the interesting suggestion.

- For journal team, a link to the "Supplementary Note 1" in the Results-Mosquitoes preparation section would be very helpful to navigate the content of the article.

Supplementary note 1 was integrated into the methods section. We have corrected this reference.

# Peer review: Reviewer response for version 2

**DOI:** 10.21956/wellcomeopenres.16821.r36163

**Published:** 2019-08-08

**Authors:** Thomas S. Churcher

**Affiliations:** 1 MRC Centre for Global Infectious Disease Analysis, Department of Infectious Disease Epidemiology, Imperial College London, London, UK

The latest version provides greater clarity. The authors did not respond why to the 13 day age group was omitted and the explanation in the text (“The age classes selected were mosquito ages 1, 3, 5, 7, 9, 11, and 15 days, which allowed acceptable per-age accuracy while improving on current binary cut-off of 4 days based on oviposition”), I don’t really understand and am a little nervous about selecting what groupings to present based on their accuracy. Nevertheless, as the authors point out this may not make a difference to the population estimate of age and they provide the raw data which will allow others to explore this should they wish. Overall the manuscript contains a substantial body of work which should form the basis of future research on the technique.

I confirm that I have read this submission and believe that I have an appropriate level of expertise to confirm that it is of an acceptable scientific standard.

# Response

**Published:** 2019-09-02

**Authors:** Klaas Wynne

**Affiliations:** University of Glasgow, UK

Thank you.

# Peer review: Reviewer response for version 1

**DOI:** 10.21956/wellcomeopenres.16586.r35616

**Published:** 2019-06-19

**Authors:** Thomas S. Churcher

**Affiliations:** 1 MRC Centre for Global Infectious Disease Analysis, Department of Infectious Disease Epidemiology, Imperial College London, London, UK

The manuscript presents the first attempt to use mid-infra-red spectroscopy to determine the age and species of Anopheles gambiae s.l mosquitoes. Novel methods for determining the age of the mosquito population are urgently needed and so the new possible technique outlined here is very much welcomed. The paper contains a great deal of information though would benefit from more clarity at times for both the methods and their rationale. Generally, the paper is written with a positive spin and the discussion could be more honest about the frailties of current results (specifically the accuracy) without diminishing the potential of the method. Conversely, the decision to classify mosquitoes into age bands instead of a continuum and the way the results are presented may make the technique look less accurate than it may well be. For example, < 20% of 3 day old *A. gambiae* mosquitoes are classified as being this age, though the confusion matrix appears to show that it is correctly identifying them as being <7 days (though it is hard to see). This level of accuracy could therefore be tolerated depending on the question under investigation, though people might look at the <20% accuracy figure and write it off.

 Specific points are detailed below. I am not qualified to comment on technical aspects of spectroscopy.

 Major points:

1. Methods. After being killed “ *Anopheles gambiae mosquitoes were kept at least one day and An. arabiensis at least two days in the vial to allow them to dry completely*.” The two species were therefore, on average, treated differently. Any conclusions about the ability of MIRS to speciate within the gambiae s.l. complex should therefore be tapered as some of the accuracy maybe because the machine learning was picking up differences in time since death/dryness?
2. Methods. Some spectra with low intensity or high water content were removed by the *Loco Mosquito *5.0 program automatically. This is available on zenodo yet no criteria for removal were given making it hard to understand what is going on. Did this preferentially exclude mosquitoes of a certain class (you can imagine water content might be related to age)?
3. Methods. Seventeen pre-selected wavelengths were used in the analysis and all other data was excluded. Why this number? It seems strange when there may be considerable information lost. Machine learning methods are designed to deal with this complexity and, though I agree about the dangers of over-fitting, this could be accounted for.
4. Methods. The machine learning methods classified mosquitoes into 7 ages classes. Why was this chosen over a continuous estimation of age? Some justification would be nice as it is different to what is typically done and indeed the authors in the results suggest there was overlap between the bands and training on daily scans (as will happen in the field) made the accuracy worse again. How was this number of classes decided upon? Previous published work has classified mosquitoes as young and old, which would have made the method look much better (it appears from the confusion matrix, it is hard to tell from the shading (consider putting numbers in). Why do those selected not cover the whole range of ages needed? Figure 16A suggests considerable numbers might be older than 15 days which would be important to the average age estimates.
5. Methods “Mosquito ages 1–15 days, taken every two days”. This does not appear to be the case as no data presented from day 13. Why have different boundaries between the oldest two classes? Is the accuracy of predicting 15 age old mosquitoes only better because there are no other classes within 4 days of them when the others have potentially 4 classes?
6. Results. The language of the results sometimes doesn’t come across as very balanced and veers off into opinion. For example, the statement “These results demonstrate that the MIRS signal is strongly indicative of mosquito species and can be used to distinguish between species in a more time and cost efficient method,” not everyone would agree with given the accuracy.

Minor points:

1. Abstract. Species accuracy is given as 82% when it only achieved an average of 76% using the steps outlined in the methods. Dropping 90 of the worst performing models gets you to the better value, but this is only done post hoc. You should either say you are going to do this in the methods or change the headline value in the abstract as it is currently confusing when the reader looks at the abstract and then Figure 13A.
2. Penultimate paragraph page 4. “16 scans were taken at room temperature”. Why was this number done and were they averaged for the spectra used in the analyses?
3. There are lots of methods in the results section. Some of this is repeated and some isn’t, the former could be deleted but the later could be transferred to the methods to aid the reader.
4. The discussion states: *“* *this method could accurately estimate the proportion of mosquitoes within the older and most epidemiologically-important age classes that responsible for malaria transmission* *”,* though mosquitoes <15 days of age are likely to be infectious. Suggest revising.
5. The discussion rather grandly says “ *To our knowledge, this is the first time that a proposed technique has had the ability to predict the age structure of a population or reconstruct demographic patterns as anticipated from specific vector control interventions*”. I’m not sure this is true, as other techniques have showed the ability but the real proof of the pudding comes when moving to the field with the considerable difference between mosquitoes (which the author highlights). Consider revising.

I confirm that I have read this submission and believe that I have an appropriate level of expertise to confirm that it is of an acceptable scientific standard, however I have significant reservations, as outlined above.

# Response

**Published:** 2019-07-30

**Authors:** Klaas Wynne

**Affiliations:** University of Glasgow, UK

Reply to reviewer comments on “Prediction of mosquito species and population age structure using mid-infrared spectroscopy and supervised machine learning [version 1; peer review: 1 approved with reservations, 1 not approved]”

Dear Dr. Churcher:

Thank you very much for taking the time to review our manuscript and for your helpful comments, which have enabled us to improve it. We detail point by point below how we addressed each remark.

*Methods. After being killed “Anopheles gambiae mosquitoes were kept at least one day and An. arabiensis at least two days in the vial to allow them to dry completely.” The two species were therefore, on average, treated differently. Any conclusions about the ability of MIRS to speciate within the gambiae s.l. complex should therefore be tapered as some of the accuracy maybe because the machine learning was picking up differences in time since death/dryness?*

Thank you very much for this comment. In the text we have tried to emphasise the fact that the *An. arabiensis* mosquitoes, because of their physiognomy, usually take one day more than *An. gambiae* to dry with silica gel. However, in our experiments all mosquitoes of both species were dried for more than three days and therefore, no significant differences in humidity that could influence the learning process are expected. We have modified the text of the methods section to clarify this point.

*Methods. Some spectra with low intensity or high water content were removed by the Loco Mosquito 5.0 program automatically. This is available on zenodo yet no criteria for removal were given making it hard to understand what is going on. Did this preferentially exclude mosquitoes of a certain class (you can imagine water content might be related to age)?*

We have now included in this section how the algorithms of "Loco mosquito" work. Since all the mosquitoes were dry, the algorithm we originally used to detect mosquitoes with high water content was no longer necessary and was removed. We have also corrected this in the methods section.

*Methods. Seventeen pre-selected wavelengths were used in the analysis and all other data was excluded. Why this number? It seems strange when there may be considerable information lost. Machine learning methods are designed to deal with this complexity and, though I agree about the dangers of over-fitting, this could be accounted for.*

The 17 wavenumbers (13 peak maxima and 4 troughs) were selected to maintain an adequate ratio between inputs and data available, with the aim to collect the maximum information from the spectra  in light of their chemical signatures without causing overfitting, a problem that had affected the previous approaches cited in our discussion. Our objective was to prove that this approach works and, as we indicated in the text, we aim to further refine the combination of MIRS and machine learning and adapt it as needed when more field data become available. We have modified the manuscript accordingly.

*Methods. The machine learning methods classified mosquitoes into 7 ages classes. Why was this chosen over a continuous estimation of age? Some justification would be nice as it is different to what is typically done and indeed the authors in the results suggest there was overlap between the bands and training on daily scans (as will happen in the field) made the accuracy worse again. How was this number of classes decided upon? Previous published work has classified mosquitoes as young and old, which would have made the method look much better (it appears from the confusion matrix, it is hard to tell from the shading (consider putting numbers in). Why do those selected not cover the whole range of ages needed? Figure 16A suggests considerable numbers might be older than 15 days which would be important to the average age estimates.*

Many thanks for this pertinent question. The age classes were selected as a compromise between granularity of the predictions and model performance. We had initially used every day (15 classes), but the models tended to perform poorly for every other age class. As mentioned in the discussion, we hypothesised that this was due to poor differentiation between subsequent days in the MIRS signals. We expect that more complex models and greater sample sizes should resolve this issue in future studies. However, it is important to note that we do not actually need day-by-day age estimates to reconstruct the age structure of the sampled mosquito population (as we show in Figure 4). This should greatly help translation of this approach to field conditions in due course.

*Methods “Mosquito ages 1–15 days, taken every two days”. This does not appear to be the case as no data presented from day 13. Why have different boundaries between the oldest two classes? Is the accuracy of predicting 15 age old mosquitoes only better because there are no other classes within 4 days of them when the others have potentially 4 classes?*

 Addressed.

*Results. The language of the results sometimes doesn’t come across as very balanced and veers off into opinion. For example, the statement “These results demonstrate that the MIRS signal is strongly indicative of mosquito species and can be used to distinguish between species in a more time and cost efficient method,” not everyone would agree with given the accuracy.*

We have reviewed results and discussion sections and corrected expressions that appear to veer off into opinion.

*Abstract. Species accuracy is given as 82% when it only achieved an average of 76% using the steps outlined in the methods. Dropping 90 of the worst performing models gets you to the better value, but this is only done post hoc. You should either say you are going to do this in the methods or change the headline value in the abstract as it is currently confusing when the reader looks at the abstract and then Figure 13A.*

Thank you very much for this comment. We indicate in the methods section that the accuracy of 82.6% is the bagged estimate from the top 10 models. The legend of Figure 13 also explains this.

*Penultimate paragraph page 4. “16 scans were taken at room temperature”. Why was this number done and were they averaged for the spectra used in the analyses?*

We have chosen to average 16 scans because this approach provides noiseless spectra in under a minute. We added more information about this in the text.

*There are lots of methods in the results section. Some of this is repeated and some isn’t, the former could be deleted but the later could be transferred to the methods to aid the reader.*

We are very grateful for this suggestion, but we consider that the results of the experiments carried out to develop our approach from scratch are relevant and, although that they could appear in the methods section, we believe they fit better in the results section given the strong element of methods development *.*

*The discussion states: “this method could accurately estimate the proportion of mosquitoes within the older and most epidemiologically-important age classes that responsible for malaria transmission”, though mosquitoes <15 days of age are likely to be infectious. Suggest revising.*

We agree that mosquitoes <15 days of age can be infectious, indeed what we suggest here is that the ability of the described approach to determine the whole-age distribution of mosquito populations (and not only individual age) is important to estimate the proportion that could be epidemiologically-important (which will vary in different settings). We have modified the text to clarify this point.

*The discussion rather grandly says “To our knowledge, this is the first time that a proposed technique has had the ability to predict the age structure of a population or reconstruct demographic patterns as anticipated from specific vector control interventions”. I’m not sure this is true, as other techniques have showed the ability but the real proof of the pudding comes when moving to the field with the considerable difference between mosquitoes (which the author highlights). Consider revising.*

We have now revised this accordingly.

# Peer review: Reviewer response for version 1

**DOI:** 10.21956/wellcomeopenres.16586.r35614

**Published:** 2019-05-31

**Authors:** Yoosook Lee

**Affiliations:** 1 Department of Pathology, Microbiology and Immunology, University of California, Davis, Davis, CA, USA

I am deeply troubled by the accuracy of the age prediction (best model was less than 60% - see Figure 3 of this article). That is not much better than flipping the coin. Species prediction is not much better with less than 80% accuracy (Figure 3). Even with bootstrapping (Figure 14), it may accurately call very young (1 day old) and very old (15 days old for *gambiae *and acceptable 80% on 11 day old *arabiensis*) but all other age groups had really poor accuracy (Figure 14). Given the low accuracy, it is hard to imagine the reliability of method as it currently stands. The current paper is written advocating this very low accuracy method. Therefore, I cannot recommend this article to others with good conscience. If the report is written in such way that discusses the limitation of this methods, then I think it is reasonable to publish. There is nothing wrong with trying and thoroughly evaluating the results.

 In the hope to improve this article, here are my suggested edits:

 In the abstract, title, or keywords there is no mention where this study is conducted. This is significant information because depending on the part of Africa, different members of *Anopheles gambiae *complex exists so the utility of testing done here distinguishing *Anopheles gambiae *from *An. arabiensis *may not transfer well in other regions where *An. gambiae s.s. *and *An. coluzzii *coexists. It is up for further study but the manuscript should be clear on the limited scope of testing done for this study to help readers contemplating using this method.

 Abstract “The accuracy of classifying mosquitoes by species was 82.6%” -> Please provide variation or standard deviation of this estimate. Also the Figure 3 data shows all less than 80% so I don’t know how the average could be 82.6%. What about the number for age prediction accuracy? Also state which model was best performing in predictions and the provide correct assignment probability for that in the abstract as author explored multiple models.

 “a negligible cost” -> provide an actual estimation in numbers (dollar or euro amount) and what’s included in the cost (equipment, labor, supplies).

 “a promising alternative to current mosquito species” -> Many would disagree that 82.6% accuracy in species diagnostic is a promising alternative. I can see the method could be a triage method for selecting specie of interest when dealing with large number of samples for genetic screening.

 First paragraph of introduction – because the method introduced here is nothing to do with insecticide resistance, this paragraph containing lengthy intro about the involvement of insecticide resistance seem unnecessary and irrelevant. The second is direct to the point of this paper and good and succinct starting point.

 “Currently, *An. gambiae *s.l. species are best distinguished by polymerase chain reaction (PCR) methods ^33,34^” -> The citation 33 is no longer valid because it doesn’t distuiguish *An. gambiae s.s. *from *An. coluzzii. *The methods by Favia *et al*. (2001) ^1^, Fanello *et al*. (2002) ^2^, and Santolamazazza *et al*. (2008) ^3^ would be more appropriate for citation here.

 “polymerase chain reaction (PCR) methods ^33,34^, which are time-consuming and expensive”. It is relative to call this method “expensive” as it would cost $2-3 US dollars to screen a single sample as opposed to Lee *et al*. (2015) ^4^ which cost $5-6 USD for a single sample but collects a wide variety of epidemiologically important traits. Of course, SNP chip or sequencing approach would cost in order of several hundreds per sample. So this molecular assays cited are on the cheap side already. Perhaps more accurate to say “which are time-consuming and still relatively costly, and can thus only be carried out on a subsample of mosquitoes collected during typical entomological surveillance conducted by many agencies in Africa”

 Method section mosquito rearing – it is not clear where the mosquito rearing occurred. Is it in Tanzania or Glasgow? Specify the location of *An. arabiensis *Ifakara strain was established.

 Method section “Mosquitoes were collected 2 days after a blood meal before egg laying (gravid) and 2 days after an oviposition cup was introduced into the cage (sugar fed).” – This part is confusing.

 “mid-infrared spectroscopy-based prediction of mosquito age structure was ….” -> Capitalize the first letter of the sentence.

 At the end of 2 ^nd^ paragraph of introduction: “…the assessment of the impact of these and other control measures”. -> It is not clear what “these and other” control measures refer to. I think it is better to drop the first paragraph and then change “these and other” to “various vector”

 “The *An. gambiae* s.l. complex includes several cryptic species that can only be distinguished by molecular analysis” - cryptic species implies two or more species hidden under one species name. I would cite the typical species diagnostic tools only dealing with known “good” species, I would use ‘sibling’ instead of ‘cryptic’.

 The introduction paragraph starting with “As in the case of age determination,..” seems out of place. 3rd and 4th paragraphs talk about age determination, 5 ^th^ paragraph talks about species determination and 6 ^th^ paragraph going back to age determination. Some rearrangement of the text necessary to make the story flow better. This paragraph is also very lengthy and makes readers wonder if there is an aging method already available for *Anopheles* species using NIRS. Rather than lengthy and detailed description of what NIRS and why it has problems, it seems the paper is better served by introducing the method of choice and its strength with appropriate citations. To be specific, I would remove the text starting with “while promising, …” till the end of 6 ^th^ paragraph.

 “Here we tested if these limitations can be overcome by shifting the measurement range (25,000-4,000 cm ^-1^) to the mid-infrared region (4,000-400 cm ^-1^), employing an attenuated total reflectance (ATR) device to assess the mosquitoes, and modelling the results with supervised machine learning.”-> In relation to the edits suggested to the previous paragraph, I would change this to “Here, we tested the id-infrared region (4,000-400 cm ^-1^), employing an attenuated total reflectance (ATR) device to assess the mosquitoes which is relatively small.” This suggested edit would make the following phrase in the sentence “Modelling the result with supervised machine learning” awkward. However, the next pagraph introduces why this approach was used so this particular phrase can be removed from this paragraph. Also any reason that the numbers are from large to small?

 Method Spectral data acquisition section - “Individual mosquitoes were laid…” -> change it to “dried specimens” to make the experimental process clearer to the readers.

 “a globar lamp” -> G should be capital on globar.

 “DLaTGS “ -> spell it out like “Deuterated Lanthanum α Alanine doped TriGlycine Sulphate (DLaTGS)”

 “KBr beamsplitter” -> spell it out like “Potassium bromide (KBr)”

 “Bruker Platinum ATR Unit A225” -> add company name and location of the company

 Method Spectral data acquisition “4,000 cm-1” -> “-1” should be superscript.

 “Loco Mosquito 5.0” – add a text like “a custom program” to indicate that this program is developed by the authors.

 Figure 1. I would prefer if the figure could be self-explanatory as much as possible without having to read legend. Add text or symbol in the figure above each sub-figures what is “correct” and “wrong” way.

 Figure 2. It is best if top panel includes an example of “good” reading to differentiate the bad readings. Also I would add text on each panel like “atmospheric intrusion”, “high water content”, and “low intensity”. Pink and red are hard to distinguish. Perhaps dotted lines for pink?

 “mod-els” -> “models”

 “suit-ed” -> “suited”

 “ma-chine” -> “machine”

I confirm that I have read this submission and believe that I have an appropriate level of expertise to state that I do not consider it to be of an acceptable scientific standard, for reasons outlined above.

## References

[1] : Molecular characterization of ribosomal DNA polymorphisms discriminating among chromosomal forms of Anopheles gambiae s.s. *Insect Mol Biol*.2001;10(1) :19-23 1124063310.1046/j.1365-2583.2001.00236.x

[2] : Simultaneous identification of species and molecular forms of the Anopheles gambiae complex by PCR-RFLP. *Med Vet Entomol*.2002;16(4) :461-4 1251090210.1046/j.1365-2915.2002.00393.x

[3] : Insertion polymorphisms of SINE200 retrotransposons within speciation islands of Anopheles gambiae molecular forms. *Malar J*.2008;7: 10.1186/1475-2875-7-163 163 10.1186/1475-2875-7-163 18724871PMC2546427

[4] : A multi-detection assay for malaria transmitting mosquitoes. *J Vis Exp*.2015; 10.3791/52385 e52385 10.3791/52385 25867057PMC4401152

# Response

**Published:** 2019-06-05

**Authors:** Klaas Wynne

**Affiliations:** University of Glasgow, UK

Dear Dr. Lee:

Thank you very much for your report. We appreciate the time you took to read our manuscript and provide constructive feedback. We believe that your major area of concern regarding the manuscript is a result of a misunderstanding about the accuracy of our method that we would now like to clarify.

In the first paragraph of your review, you stated:

*I am deeply troubled by the accuracy of the age prediction (best model was less than 60% - see Figure 3 of this article). That is not much better than flipping the coin. Species prediction is not much better with less than 80% accuracy (Figure 3). Even with bootstrapping (Figure 14), it may accurately call very young (1 day old) and very old (15 days old for gambiae and acceptable 80% on 11 day old arabiensis) but all other age groups had really poor accuracy (Figure 14). Given the low accuracy, it is hard to imagine the reliability of method as it currently stands.*

As we indicated in the paper (page 5, version 1), Figure 3 represents a comparison between different algorithms using default (i.e., non-optimised) settings. We do not claim nor suggest that these represent our ‘best models’, which don’t appear in the paper until Figure 13. Indeed, our best models achieved 82.6%, not 60% accuracy.

Because the age model is predicting one of 7 balanced age classes, the null expectation at random is 14.3% accuracy, not a coin flip which is 50%. Consequently, our models need only exceed 14.3% to be better than chance alone. In other words, this is a multi-class rather than binary classification model.

To avoid confusion, we have indicated in figures 13A, 14A, and 14C the accuracy expected under chance alone (red horizontal dashed line) and further clarified the difference between Figures 3 and 13/14 in the text and the caption of Figure 3.

We would like to thank you again for the suggestions to improve our manuscript. This bullet point list shows how we have addressed them.

- *In the abstract, title, or keywords there is no mention where this study is conducted. This is significant information because depending on the part of Africa, different members of Anopheles gambiae complex exists so the utility of testing done here distinguishing Anopheles gambiae from An. arabiensis may not transfer well in other regions where An. gambiae s.s. and An. coluzzii coexists. It is up for further study but the manuscript should be clear on the limited scope of testing done for this study to help readers contemplating using this method.*

We thank the reviewer for this suggestion. To address your concern, we now explicitly indicated in the abstract that the work was developed using laboratory colonies and specified that further study is needed to test this approach on wild populations. Also, in the keywords the two mosquito species are now indicated as Anopheles gambiae s.s. and Anopheles arabiensis, to avoid any ambiguity.

- *Abstract “The accuracy of classifying mosquitoes by species was 82.6%” -> Please provide variation or standard deviation of this estimate. *

The accuracy of 82.6% is the bagged estimate from the top 10 models. The standard deviation on those 10 models is ± 0.002%, but all that says is that the 10 models are all very similar to each other. Therefore, we believe that this number is not very useful and have elected not to include this number.

- *Also the Figure 3 data shows all less than 80% so I don’t know how the average could be 82.6%. What about the number for age prediction accuracy? Also state which model was best performing in predictions and the provide correct assignment probability for that in the abstract as author explored multiple models*

Please see response to general points above.

- *“a negligible cost” -> provide an actual estimation in numbers (dollar or euro amount) and what’s included in the cost (equipment, labor, supplies).*

Page 17 includes a description of the cost of our method.

- *“a promising alternative to current mosquito species” -> Many would disagree that 82.6% accuracy in species diagnostic is a promising alternative. I can see the method could be a triage method for selecting specie of interest when dealing with large number of samples for genetic screening.*

We believe it is a promising alternative. This paper represents an initial assessment that shows that machine learning can be used to predict traits from the complex infrared spectra of mosquitoes’ cuticles and that it is possible to develop a method to survey mosquitos efficiently and with a negligible cost. We believe that this is only the beginning as the evidence presented in the manuscript strongly suggests that this approach is amenable to vast improvements as more field data become available.

- *First paragraph of introduction – because the method introduced here is nothing to do with insecticide resistance, this paragraph containing lengthy intro about the involvement of insecticide resistance seem unnecessary and irrelevant. The second is direct to the point of this paper and good and succinct starting point.*

We believe this first paragraph is important as it introduces the current context (increased insecticide resistance in malaria vector populations) where monitoring mosquito species and age structure is becoming essential to assess the effectiveness of control tool. Without this first paragraph, this context would be lost, so we politely disagree with this point.

- *“Currently, An. gambiae s.l. species are best distinguished by polymerase chain reaction (PCR) methods 33,34” -> The citation 33 is no longer valid because it doesn’t distuiguish An. gambiae s.s. from An. coluzzii. The methods by Favia et al. (2001) 1, Fanello et al. (2002) 2, and Santolamazazza et al. (2008) 3 would be more appropriate for citation here.*

Suggestion attended to. Thank you very much for these references.

- *“polymerase chain reaction (PCR) methods33,34, which are time-consuming and expensive”. It is relative to call this method “expensive” as it would cost $2-3 US dollars to screen a single sample as opposed to Lee et al. (2015) 4 which cost $5-6 USD for a single sample but collects a wide variety of epidemiologically important traits. Of course, SNP chip or sequencing approach would cost in order of several hundreds per sample. So this molecular assays cited are on the cheap side already. Perhaps more accurate to say “which are time-consuming and still relatively costly, and can thus only be carried out on a subsample of mosquitoes collected during typical entomological surveillance conducted by many agencies in Africa”*

Suggestion attended to.

- *Method section mosquito rearing – it is not clear where the mosquito rearing occurred. Is it in Tanzania or Glasgow? Specify the location of An. arabiensis Ifakara strain was established.*

We have now specified that mosquito rearing has occurred at the University of Glasgow; we also indicated that An. arabiensis Ifakara strain was initially established in Tanzania at the Ifakara Health Institute and then also reared at the University of Glasgow.

- *Method section “Mosquitoes were collected 2 days after a blood meal before egg laying (gravid) and 2 days after an oviposition cup was introduced into the cage (sugar fed).” – This part is confusing.*

We have now modified this section to make it clearer. It now reads: “Mosquitoes under three types of physiological conditions were collected, specifically: mosquitoes that had just received a blood meal (blood fed), mosquitoes that had developed eggs as they received a blood meal two days before collection (gravid), and mosquitoes that had laid eggs as they received a blood meal four days before collection and had the chance to lay eggs on an oviposition cup for two consecutive nights (sugar fed).”

- *“mid-infrared spectroscopy-based prediction of mosquito age structure was ….” -> Capitalize the first letter of the sentence.*

Suggestion attended to.

- *At the end of 2nd paragraph of introduction: “…the assessment of the impact of these and other control measures”. -> It is not clear what “these and other” control measures refer to. I think it is better to drop the first paragraph and then change “these and other” to “various vector”.*

We have changed the paragraph to include your suggestion. Thank you very much.

- *“The An. gambiae s.l. complex includes several cryptic species that can only be distinguished by molecular analysis” - cryptic species implies two or more species hidden under one species name. I would cite the typical species diagnostic tools only dealing with known “good” species, I would use ‘sibling’ instead of ‘cryptic’.*

We have now removed the adjective cryptic which can be misleading and used sibling species instead.

- *The introduction paragraph starting with “As in the case of age determination,..” seems out of place. 3rd and 4th paragraphs talk about age determination, 5th paragraph talks about species determination and 6th paragraph going back to age determination. Some rearrangement of the text necessary to make the story flow better. This paragraph is also very lengthy and makes readers wonder if there is an aging method already available for Anopheles species using NIRS. Rather than lengthy and detailed description of what NIRS and why it has problems, it seems the paper is better served by introducing the method of choice and its strength with appropriate citations. To be specific, I would remove the text starting with “while promising, …” till the end of 6th paragraph.*

We have modified the indicated paragraphs following your suggestions to facilitate the reading. However, we have not removed the part about near infrared spectroscopy. We believe that given the similarity at first sight between NIRS techniques and our approach, we should clarify from the introduction why our method is novel and what are its advantages.

- *“Here we tested if these limitations can be overcome by shifting the measurement range (25,000-4,000 cm-1) to the mid-infrared region (4,000-400 cm-1), employing an attenuated total reflectance (ATR) device to assess the mosquitoes, and modelling the results with supervised machine learning.”-> In relation to the edits suggested to the previous paragraph, I would change this to “Here, we tested the id-infrared region (4,000-400 cm-1), employing an attenuated total reflectance (ATR) device to assess the mosquitoes which is relatively small.” This suggested edit would make the following phrase in the sentence “Modelling the result with supervised machine learning” awkward. However, the next pagraph introduces why this approach was used so this particular phrase can be removed from this paragraph.*

Please see above.

- *Also any reason that the numbers are from large to small?*

It is customary among IR spectroscopists to use wavenumbers as energy unit and express it from higher to lower (because it is the reciprocal unit of wavelength, the unit that was traditionally used).

- *Method Spectral data acquisition section - “Individual mosquitoes were laid…” -> change it to “dried specimens” to make the experimental process clearer to the readers.*

Suggestion attended to.

- *“a globar lamp” -> G should be capital on globar.*

Suggestion attended to.

- *“DLaTGS “ -> spell it out like “Deuterated Lanthanum α Alanine doped TriGlycine Sulphate (DLaTGS)”*

Suggestion attended to.

- *“KBr beamsplitter” -> spell it out like “b”*

Suggestion attended to.

- *“Bruker Platinum ATR Unit A225” -> add company name and location of the company*

Suggestion attended to.

- *Method Spectral data acquisition “4,000 cm-1” -> “-1” should be superscript.*

Suggestion attended to.

- *“Loco Mosquito 5.0” – add a text like “a custom program” to indicate that this program is developed by the authors.*

Suggestion attended to.

- *Figure 1. I would prefer if the figure could be self-explanatory as much as possible without having to read legend. Add text or symbol in the figure above each sub-figures what is “correct” and “wrong” way.*

Suggestion attended to. We have included in the figure a green check mark and a red cross mark.

- *Figure 2. It is best if top panel includes an example of “good” reading to differentiate the bad readings. Also I would add text on each panel like “atmospheric intrusion”, “high water content”, and “low intensity”. Pink and red are hard to distinguish. Perhaps dotted lines for pink?*

Suggestion attended to. Labels added and pink line substituted by a black dashed line.

- *“mod-els” -> “models”*

Suggestion attended to.

- *“suit-ed” -> “suited”*

Suggestion attended to.

- *“ma-chine” -> “machine”*

Suggestion attended to. We really appreciate your effort to detect all these typos.

Regarding your concern about the availability of our data expressed in the final questions to the reviewer, we have noticed during the editing of our manuscript the DOI link to our data has changed. We are going to ask the editors to restore the original link (https://doi.org/10.5525/gla.researchdata.688)
